# Impacts of performance-based financing on health system performance: evidence from the Democratic Republic of Congo

**DOI:** 10.1186/s12916-023-03062-8

**Published:** 2023-10-04

**Authors:** Gil Shapira, Emma Clarke-Deelder, Baudouin Makuma Booto, Hadia Samaha, György Bèla Fritsche, Michel Muvudi, Dominique Baabo, Delphin Antwisi, Didier Ramanana, Saloua Benami, Günther Fink

**Affiliations:** 1 The World Bank, Washington, D.C. USA; 2 The Swiss Tropical and Public Health Institute and University of Basel, Basel, Switzerland; 3 Ministry of Public Health, Kinshasa, Democratic Republic of Congo

**Keywords:** Performance-based financing, Health systems, Quality of care, Maternal and child health

## Abstract

**Background:**

Health systems’ weakness remains one of the primary obstacles towards achieving universal access to quality healthcare in low-income settings. Performance-based financing (PBF) programs have been increasingly used to increase access to quality care in LMICs. However, evidence on the impacts of these programs remains fragmented and inconclusive. We analyze the health system impacts of the PBF program in the Democratic Republic of the Congo (DRC), one of the largest such programs introduced in LMICs to date.

**Methods:**

We used a health systems perspective to analyze the benefits of PBF relative to unconditional financing of health facilities. Fifty-eight health zones in six provinces were randomly assigned to either a control group (28 zones) in which facilities received unconditional transfers or to a PBF program (30 zones) that started at the end of 2016. Follow-up data collection took place in 2021–2022 and included health facility assessments, health worker interviews, direct observations of consultations and deliveries, patient exit interviews, and household surveys. Using multivariate regression models, we estimated the impact of the program on 55 outcomes in seven health system domains: structural quality, technical process quality, non-technical process quality, service fees, facility management, providers’ satisfaction, and service coverage. We used random-effects meta-analysis to generate pooled average estimates within each domain.

**Results:**

The PBF program improved the structural quality of health facilities by 4 percentage points (ppts) (95% CI 0.01–0.08), technical process quality by 5 ppts (0.03–0.07), and non-technical process by 2 ppts (0–0.04). PBF also increased coverage of priority health services by 3 ppts (0.02–0.04). Improvements were also observed for facility management (9 ppts, 0.04–0.15), service fee policies, and users’ satisfaction with service affordability (14 ppts, 0.07–0.20). Service fees and health workers’ satisfaction were not affected by the program.

**Conclusions:**

The results suggest that well-designed PBF programs can lead to improvements in most health systems domains relative to comparable unconditional financing. However, the large persisting gaps suggest that additional changes, such as allocating more resources to the health system and reforming the human resources for health management, will be necessary in DRC to achieve the ambitious global universal health coverage and mortality goals.

**Trial registration:**

American Economics Association Trial registry AEARCTR-0002880.

**Supplementary Information:**

The online version contains supplementary material available at 10.1186/s12916-023-03062-8.

## Background

Although coverage of essential health services has increased in recent decades in low- and middle-income countries, the burden of preventable mortality remains high and appears to be driven largely by lack of access to quality care [1]. In many health facilities, major quality gaps have been observed even for basic services such as routine pediatric and antenatal care [2, 3]. At the same time, the share of the population incurring financial hardship associated with the use of health services has increased and inequities with respect to service access and health outcomes persist [4]. Facing these challenges, countries have increasingly turned to pay-for-performance approaches to reform their health systems [5]. The objective of these approaches is to use scarce resources more efficiently by incentivizing high-priority, high-quality, and cost-effective services, including for hard-to-reach groups, while encouraging providers to reduce fees [6].

Performance-based financing (PBF) is a type of pay-for-performance approach that provides financial incentives to health facilities based on quantity of services, adjusted for quality of care [7]. Following the positive results of an early PBF program in Rwanda [8], similar programs were implemented in several other low- and lower-middle-income countries. Impact evaluations of some of these programs, however, yielded highly heterogeneous results [9–15]. While PBF programs comprise a rather complex set of interventions essentially affecting all aspects of health systems, most studies report results on a limited set of outcomes. Given the variations in program designs, heterogeneity in results, and narrow focus of most past studies, the currently available evidence makes it difficult to assess the overall desirability of these programs from a policy perspective.

In this study, we used a health systems perspective to analyze one of the largest PBF programs implemented to date in terms of the population benefitting from the program. The PBF program in the Democratic Republic of Congo (DRC) was launched in 2016 with the objective of improving the quality and quantity of health services delivered by primary care health centers and hospitals for a population of about 30 million in eleven provinces. To allow a rigorous assessment of the program, 58 health zones in six provinces were selected for a comprehensive impact evaluation and a large survey and surveillance program implemented at the facility and household level across all 58 zones. Thirty of the evaluation zones were randomly selected for the PBF program. Facilities in control health zones received matching unconditional cash grants, thus allowing us to measure the health system impact of the PBF mechanisms keeping average resources available to facilities the same. In this paper, we first present a stylized theory of change and then evaluate the impact of the program on a comprehensive set of outcomes across seven health system domains: structural quality of health facilities, technical and non-technical process quality, service fees, facility management, health worker satisfaction, and service coverage.

## Methods

### Setting

With an estimated maternal mortality rate of 473 per 100,000 live births [16] and an under-5 mortality of 81 per 1000 births (World Development Indicators 2022), the DRC remains among the countries with the poorest health indicators globally. Seventy percent of the population, currently estimated at 96 million, lives below the international poverty line of 2.15 USD a day (World Development Indicators 2022). Even though a relatively high share of women receives at least some antenatal care (82%) and delivers in health facilities (82%), major gaps in other areas such as vaccination coverage and quality of health services remain [17–21].

In terms of the health system, the country’s 26 provinces are divided into 516 health zones that typically comprise a single first-level referral hospital and 12–20 health centers; each health center is responsible for a catchment area of about 10,000. The current health expenditure per capita was 21 USD in 2020, only about half of the 39 USD low-income country average. Central government health spending amounts to only 16% of the total health expenditure (Global Health Expenditure Database). It covers a small share of health workers’ income and most health facilities do not routinely receive financing or other resources. As a result, health facilities, whether public or private, heavily rely on user fees to remunerate their staff, procure supplies, and cover other operating costs [22].

The study presented here was conducted in 58 health zones across six provinces covered by a World Bank-financed project. The provinces are Kwango, Kwilu, and Mai-Ndombe in the west of DRC and Haut Katanga, Haut Lomami, and Lualaba in the southeast. Additional information on the zone selection is presented in Additional file 1.

### The performance-based financing scheme

The DRC PBF program has been implemented by the Ministry of Public Health since 2016 with financing from the World Bank as part of the larger Health System Strengthening Project. The program offered contracted health facilities quarterly payments conditional on the volumes of primarily reproductive, maternal, and child health services. A complete list of services, fee scales, and additional information on the program design are presented in Additional file 1: Table S1-S2.

In addition to the quantity-based incentives, facilities also received quality-based payments based on a detailed quality checklist. As shown in the Additional file 1: Tables S3-S4, the checklists contained a range of indicators related to both structural and process quality [23] and were completed primarily through review of documents and registries, and verification of the availability of different supplies. The quality bonus was proportional to the quarterly rewarded for the number of incentivized services provided (quantity bonus). Facilities that scored less than 50% on the quality checklist did not receive any quality bonus. Health centers could receive a maximum bonus of 25% of quantity bonus, while hospitals could get a maximum bonus of 40% of the total quantity-based transfers made.

The financing received through the intervention, equaling approximately $1.6 USD per capita per year, did not replace any source of funding previously received by the facilities. However, the program introduced rules for how contracted facilities use their revenues, whether received as PBF payments or through any other source. Facilities were allowed to spend a maximum of 50% of each quarter’s revenue on personal bonuses for staff. The amount received by each staff member depended mostly on individual quarterly performance evaluations based on an evaluation tool developed by the project and to a lesser extent on grade, title, and seniority. The rest of the budget needed to be allocated based on a quarterly business plan, which facility managers developed using another structured tool developed for the project. Since 2020, facilities were required to spend a minimum of 20% of the PBF payments on medications and other consumables.

A detailed data reporting verification and counter-verification system, involving both reviews of facility registers, and tracking of users at the community level, was set up to minimize the risk of erroneous or fraudulent reporting. In addition, health zone teams were incentivized to conduct routine supervision and coaching of health facilities, including assistance in the elaboration of the quarterly business plans.

### Experimental design

Out of the 58 evaluation health zones, 30 were selected for the PBF program through public randomization ceremonies conducted in each province in the presence of representatives from all health zones. The randomization was blocked by province. Additional file 1: Figure S1 shows the spatial location of the zones and the randomization results.

In the control health zones, facilities received quarterly transfers equaling the average transfer made to facilities in the PBF zones in the same province, adjusted for the population in the catchment areas and equity classification. There was no verification of service volumes reported by facilities in the control zones, and there was no assessment of their quality with the quality checklist. These facilities also did not need to comply with the program rules regarding spending their revenues and allocation of staff bonuses. However, control facilities also had to spend a minimum of 20% of their transfers on medications since 2020.

Facilities in both treatment arms received an initial investment transfer at the beginning of the project’s implementation as well as a one-off shipment of medications and family planning products. The unconditional financing began at the same time as the PBF program was launched in each province: the last quarter of 2016 in the provinces Kwango, Kwilu, and Mai-Ndombe and in the third quarter of 2017 in the other three provinces.

### Data collection

**Fig. 1:**
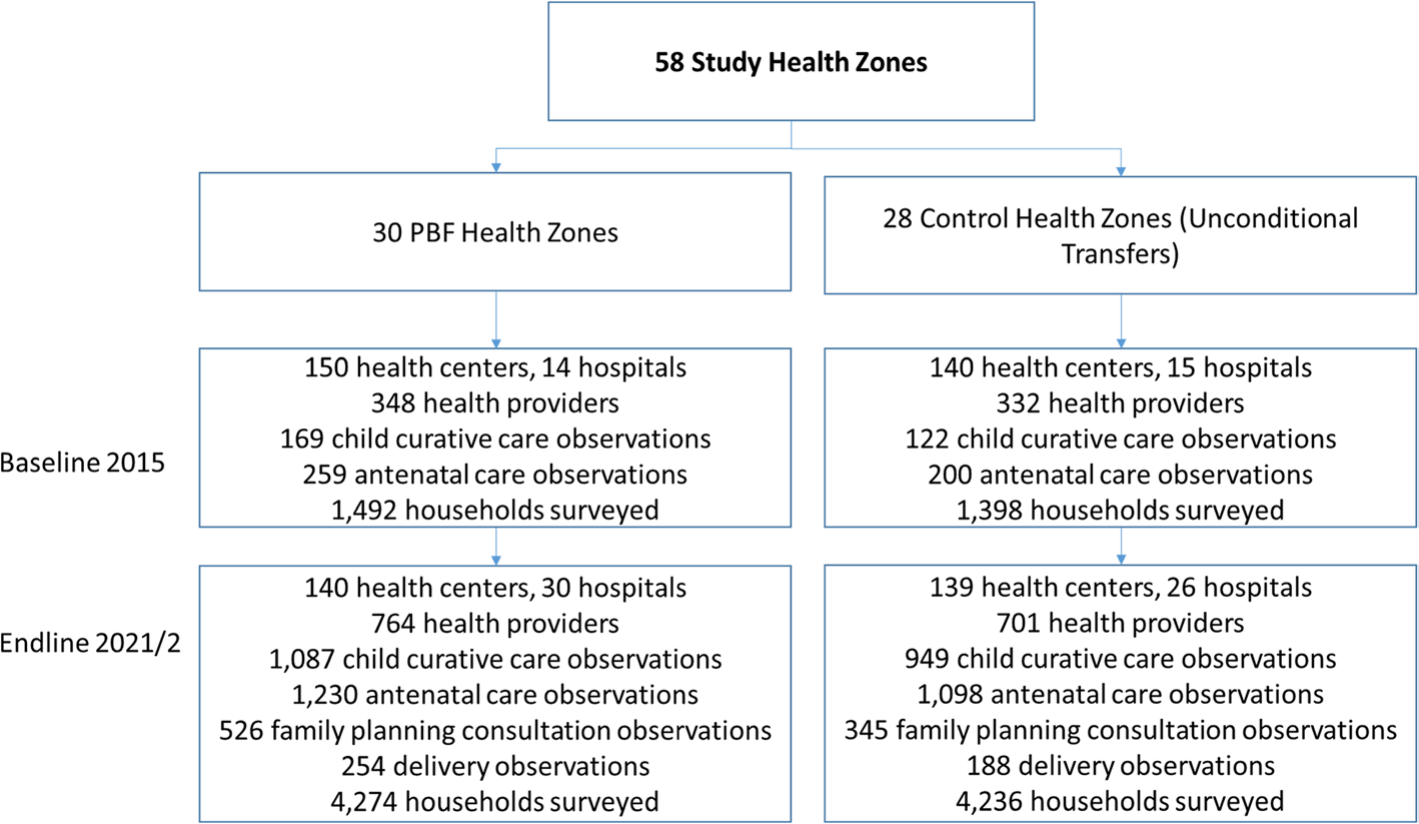
Study design. The results presented in the paper are only of the follow-up survey data. In Additional file 1, we show an analysis of the baseline data to test for balance between the control and the PBF groups

To allow a comprehensive assessment of program impact, two major rounds of data collection at the facility and household level were conducted: a first round in 2015 (baseline), and a second round in 2021 (endline). In the first step, five health centers were randomly selected together with the main referral hospital within each health zone for an in-depth health facility assessment. The health facility assessments included a general facility survey completed by the facility-in-charge; interviews with health providers; direct observations of under-5 outpatient consultations, ANC visits, family planning consultations, and deliveries; and patient exit interviews. This facility-based data was complemented with data from interviews with a representative set of households in the catchment area of each facility. To identify households, the research team first identified all villages (or neighborhoods) in the catchment area of each facility and then randomly selected one village for the survey. Within each selected village, all households were listed by the survey team. During the baseline, 10 women with current or recent pregnancies were selected for an interview in each village. During the follow-up, 27 women between the ages of 15 and 49 were selected per village for an interview, including women without recent pregnancies. Figure 1 summarizes the overall study design and Additional file 1 provides additional information on the data collection, sampling framework, and power calculations.

### Theoretical framework

**Fig. 2:**
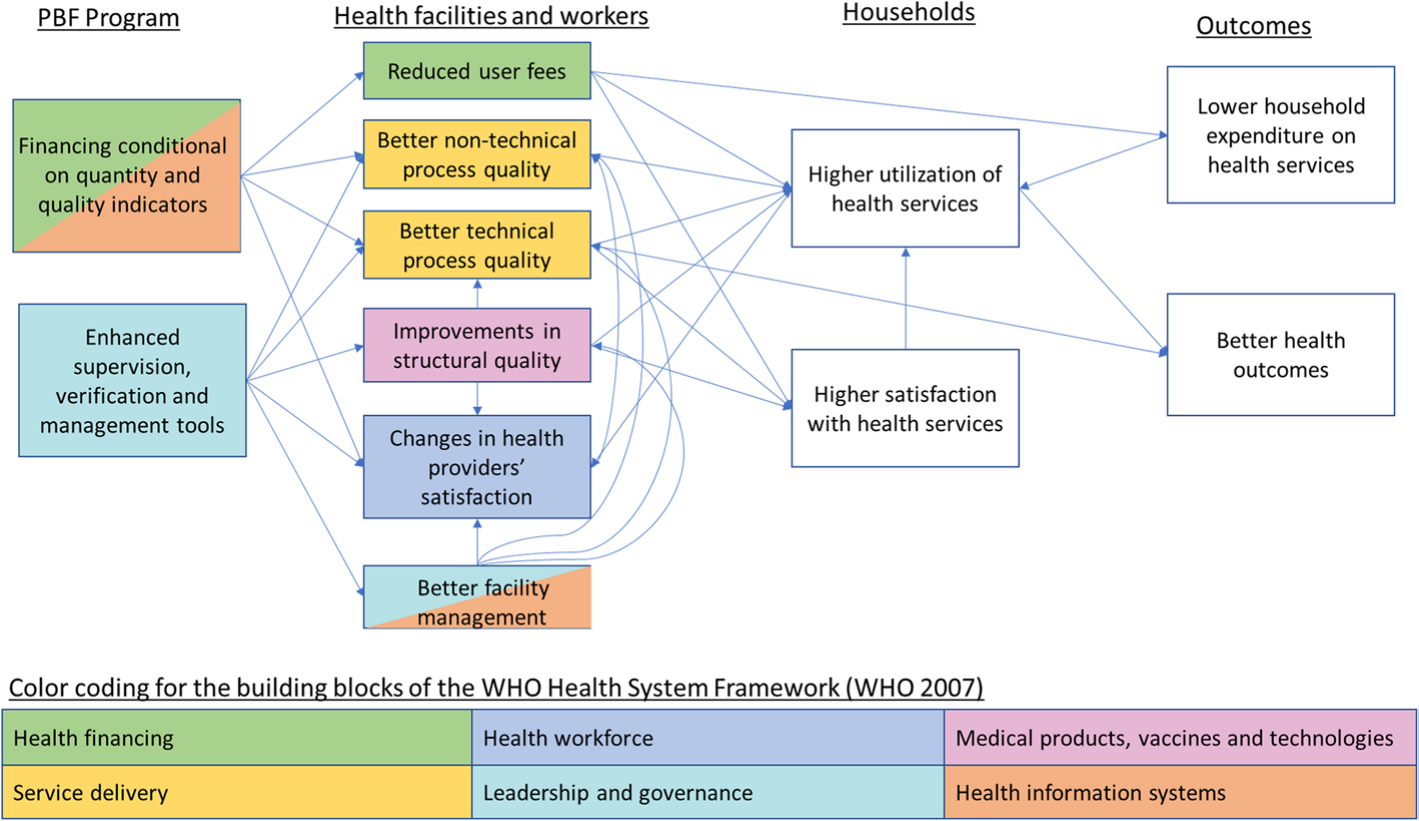
Theory of change

Figure 2 presents the theory of change underlining the expected changes in the PBF arm relative to the control arm receiving the unconditional transfers. The figure also illustrates the mechanisms through which PBF is hypothesized to improve outcomes relate to all six building blocks of the World Health Organization (WHO) Health System Framework [24]. In response to the conditionality of the financial incentives, together with the enhanced supervision and verification, facilities are expected to improve the quality of care, which should attract more patients over time. Following the Donabedian framework [23], improvement can occur in terms of structural or process quality. Structural quality mostly captures service readiness while process quality captures the technical (clinical) and non-technical aspects of patient-provider interactions. Facilities are also expected to reduce user fees to further increase demand for their services. The supervision and verification, together with the management tools (quarterly business plan and staff evaluation tool) are expected to improve facility management; this could further contribute to quality improvements through improved decisions about resource allocations and coaching and monitoring of staff. Observing improved quality of care and reduced user fees, households are expected to use more services while paying less per service and to have higher satisfaction with health facilities.

It is theoretically ambiguous how the PBF program would affect staff satisfaction, given that by construction unconditional facility financing was set up to ensure equal financial resources would be available to facilities in both groups. Changes in the within-facility distribution of staff remuneration were of course possible. Intrinsically motivated providers could also react positively to the better structural quality as well as the more structured supervisions that can enable improvements in competencies. On the other hand, higher patient volumes and enhanced monitoring and reporting requirements could increase provider workload and reduce their job satisfaction.

### Variables and outcome measures

We divided our outcome measures into seven domains included in the theory of change presented in Fig. 2. We considered three domains of service quality: structural quality, technical process quality, and non-technical process quality. Non-technical process quality combined measures of respectful care reported by enumerators observing consultations and deliveries, and users’ satisfaction reported during exit interviews. For service fees, we analyzed both the official service fees reported by facilities and payments reported by users during the exit interviews. We also analyzed binary measures related to fee policies and users’ satisfaction with service affordability. Providers’ satisfaction was measured through a series of work satisfaction questions directly asked to providers. Service coverage measures were based on household survey self-reports. In total, we analyzed 55 indicators in 8 groups, with many indicators calculated as indexes summarizing an even larger set of indicators. Detailed definitions of all indicators analyzed as well as their data sources are presented in Additional file 1.

### Statistical analysis

We used multivariate linear regression models to estimate the impact of the PBF treatment on each outcome. All models controlled for the randomization block (province). For outcomes related to directly observed consultations, we controlled for facility, provider, and user characteristics. Standard errors were clustered at the health zone level – the level at which the PBF program was allocated. Additional information on the covariates and power calculation is presented in Additional file 1. Given the large number of outcomes analyzed, we used random-effects meta-analysis to generate a pooled average estimate within each domain. The main assumption underlying the random effects meta-analysis approach is that the true causal effects of an intervention are distributed normally around a central mean effect, which we believe is a reasonable assumption within each health systems domain. All statistical analysis was conducted using the Stata SE 16.0 software package.

## Results

**Fig. 3:**
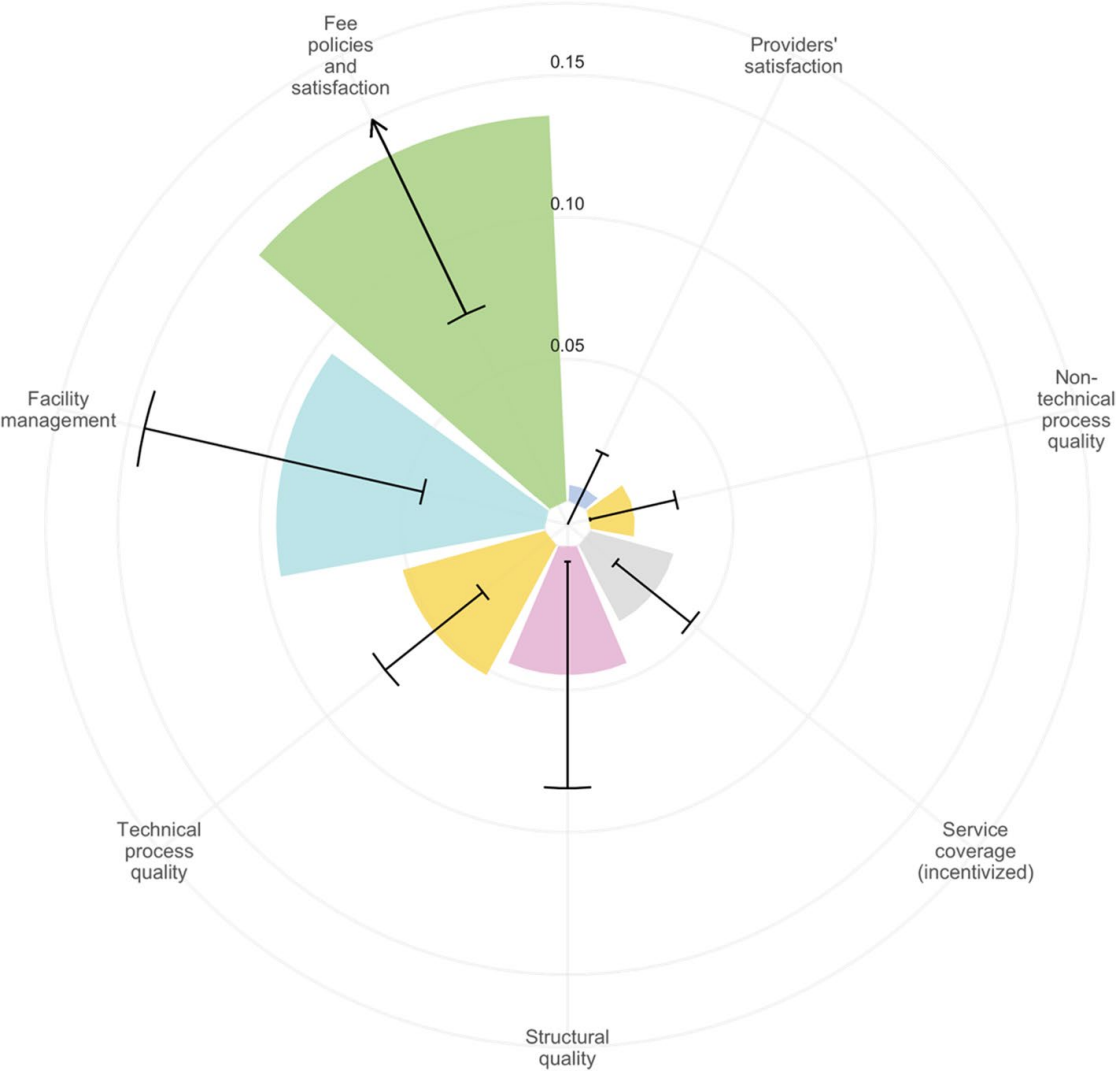
Domain-specific average impact. The meta-analysis results are presented separately for each domain, pooling data from health centers and hospitals; colored bars indicate impact estimates from random effects meta-analysis in each domain. Colors correspond with the colors used for WHO building blocks in Fig. 2. Black error bars indicate 95% confidence intervals. Forest plots with the weight assigned to each indicator are presented in Additional file 1: Figures S2-S8

The effects of the PBF program on individual indicators are presented in Tables 1 and 2, with impact results presented separately for health centers and hospitals. Unless otherwise stated, the text below describes the results of the meta-analysis, shown in Fig. 3, that pools the data from the health centers and hospitals when applicable Additional results are presented in Additional file 1.

**Table 1:** Effects of the PBF program on outcomes at the health facility level

Variable	Health centers				Hospitals			
	**Mean in control group**	**Impact and CI**	**% Change**^**b**^	***N***	**Mean in control group**	**Impact and CI**	**% Change**^**b**^	***N***
**Panel a: structural quality**								
Basic equipment index	0.54	0.04 (0–0.07)	7%	290	0.75	0.01 (− 0.03 to 0.05)	1%	56
Essential medicine and consumables index	0.68	0.01 (− 0.04 to 0.07)	2%	290	0.85	− 0.04 (− 0.11 to 0.03)	− 5%	56
Vaccines index	0.62	0.05 (− 0.08 to 0.17)	8%	275	0.13	− 0.06 (− 0.2 to 0.09)	− 47%	53
Family planning products index	0.35	0.19 (0.11–0.27)	55%	290	0.53	0 (− 0.12 to 0.12)	− 1%	56
Infrastructure index	0.36	0.02 (− 0.03 to 0.07)	6%	290	0.65	0.04 (− 0.05 to 0.14)	7%	56
Infection prevention and control index	0.45	0.04 (− 0.03 to 0.11)	9%	264	0.75	0.01 (− 0.11 to 0.13)	2%	55
**Average impact**^**a**^	0.50	**0.05 (0.01–0.09)**	10%		0.72	**0 (**− **0.03 to 0.03)**	0%	
**Panel b: technical process quality**								
Antenatal care score	0.91	0.02 (− 0.03 to 0.08)	3%	1482	0.87	0.06 (0–0.13)	7%	283
IMCI: assessment score	0.56	0.03 (− 0.01 to 0.08)	6%	1631	0.65	0.05 (− 0.01 to 0.1)	7%	364
IMCI: diagnosis score	0.68	0.05 (− 0.02 to 0.12)	7%	1240	0.59	0.12 (− 0.04 to 0.28)	21%	272
IMCI: correct treatment	0.64	0.06 (− 0.04 to 0.16)	9%	1304	0.64	− 0.06 (− 0.17 to 0.06)	− 9%	294
IMCI: no unnecessary treatment	0.60	0.07 (− 0.05 to 0.19)	11%	422	0.52	− 0.04 (− 0.31 to 0.22)	− 8%	74
Family planning consultation score	0.67	0.15 (0.06–0.23)	16%	575	0.89	0.06 (− 0.04 to 0.16)	7%	159
Delivery score	0.60	0.03 (− 0.04 to 0.1)	5%	259	0.67	0.07 (− 0.01 to 0.15)	10%	135
Postpartum care score	0.07	0.03 (− 0.05 to 0.12)	45%	278	0.02	0.17 (0.02–0.31)	1063%	141
Newborn care score	0.74	0.04 (− 0.08 to 0.16)	6%	279	0.76	0.11 (− 0.01 to 0.22)	14%	143
**Average impact**^**a**^	0.55	**0.04 (0.02**–**0.07)**	7%		0.62	**0.06 (0.03**–**0.09)**	10%	
**Panel c: non-technical process quality**								
Antenatal care respect index	0.80	− 0.03 (− 0.1 to 0.03)	− 4%	1489	0.81	0.03 (− 0.1 to 0.15)	3%	283
Antenatal care user satisfaction	0.91	0.01 (− 0.02 to 0.04)	1%	1475	0.87	− 0.01 (− 0.09 to 0.07)	− 1%	281
Child curative care respect index	0.75	0.01 (− 0.07 to 0.1)	2%	1631	0.76	0.08 (− 0.01 to 0.17)	10%	364
Child curative care user satisfaction	0.88	0 (− 0.04 to 0.04)	0%	1610	0.83	0.01 (− 0.07 to 0.09)	1%	362
Family planning consultation respect index	0.82	0.09 (0.03–0.16)	12%	575	0.81	0.05 (− 0.06 to 0.16)	6%	159
Family planning consultation user satisfaction	0.91	0.01 (− 0.04 to 0.06)	1%	563	0.89	0.01 (− 0.06 to 0.07)	1%	155
Delivery care respect index	0.96	0.04 (0–0.08)	4%	294	0.95	0.01 (− 0.05 to 0.06)	1%	148
**Average impact**^**a**^	0.88	**0.02 (**− **0.01 to 0.04)**	2%		0.88	**0.02 (**− **0.01 to 0.05)**	2%	
**Panel d: service fees (in Congolese Francs)**								
ANC fee reported by facility	2493	− 720 (− 1270 to − 170)	− 29%	289	2540	425 (− 1402 to 2252)	17%	54
Delivery fee reported by facility	8734	− 1733 (− 3606 to 141)	− 20%	288	11,788	− 1959 (− 5764 to 1847)	− 17%	56
Family planning consultation fee reported by facility	574	− 198 (− 524 to 128)	− 34%	228	1000	− 545 (− 1472 to 382)	− 54%	52
Curative care fee reported by facility (not specific for child care)	3120	638 (− 37 to 1313)	20%	244	4438	− 567 (− 4104 to 2970)	− 13%	16
ANC fees reported in exit interviews	1976	149 (− 660 to 958)	8%	1489	3221	1098 (− 944 to 3139)	34%	283
Child curative care fees reported in exit interviews	4997	− 1006 (− 1897 to − 116)	− 20%	1631	13,917	− 1238 (− 4460 to 1984)	− 9%	364
Family planning consultation fee reported in exit interviews	704	− 30 (− 608 to 548)	− 4%	577	1980	− 819 (− 2531 to 892)	− 41%	159
**Average impact**^**a**^	2,355	− **248 (**− **657 to 162)**	− **11%**		2,574	− **330 (**− **973 to 314)**	− 13%	
**Panel e: fee policies and user satisfaction with fees**								
Flat fees	0.51	0.22 (0.08–0.36)	43%	290	0.58	0.14 (− 0.13 to 0.4)	24%	56
Fees posted	0.63	0.17 (0.06–0.28)	27%	290	0.69	0.29 (0.09–0.48)	41%	56
Fee exemptions for poor users	0.67	0.19 (0.1–0.29)	29%	287	0.77	0.05 (− 0.16 to 0.27)	7%	56
Satisfaction with service affordability in antenatal care exit interviews	0.91	0.11 (0.01–0.2)	12%	1266	0.87	0.17 (0.05–0.3)	20%	259
Satisfaction with service affordability in child care exit interviews	0.88	0.03 (− 0.03 to 0.08)	3%	1560	0.83	0.03 (− 0.15 to 0.2)	3%	359
Satisfaction with service affordability in family planning exit interviews	0.91	0.12 (− 0.05 to 0.29)	13%	245	0.89	0.19 (− 0.05 to 0.44)	22%	88
**Average impact**^**a**^	0.70	**0.13 (0.06**–**0.20)**	18%		0.67	**0.15 (0.08**–**0.23)**	22%	
**Panel f: facility management**								
Protocols index	0.43	0.11 (0.03–0.19)	25%	290	0.66	0.08 (− 0.02 to 0.19)	13%	56
Reporting index	0.76	0.06 (0–0.12)	8%	197	0.93	0.04 (− 0.03 to 0.11)	4%	39
Posting of infection and control procedures index	0.25	0.19 (0.06–0.32)	76%	264	0.54	0.26 (0.07–0.45)	48%	55
Mechanism to Seek patients’ opinions	0.60	0.07 (− 0.07 to 0.21)	12%	290	0.54	0.22 (− 0.02 to 0.46)	41%	56
**Average impact**^**a**^	0.57	**0.09 (0.04**–**0.15)**	16%		0.73	**0.11 (0.02**–**0.21)**	15%	
**Panel g: providers’ satisfaction**								
Satisfied with information on own performance	0.77	0.02 (− 0.06 to 0.1)	3%	1129	0.64	0.09 (− 0.07 to 0.25)	14%	275
Satisfied with level of autonomy	0.79	− 0.05 (− 0.12 to 0.02)	− 6%	1146	0.74	0 (− 0.12 to 0.12)	0%	278
Satisfied with the relationship with the local facility committee	0.74	0.01 (− 0.05 to 0.07)	1%	1019	0.66	0.08 (− 0.04 to 0.21)	13%	225
Satisfied with support from supervisor	0.75	0.03 (− 0.03 to 0.1)	4%	1153	0.71	0.09 (− 0.04 to 0.22)	12%	274
Satisfied with recognition received from supervisor	0.79	0.02 (− 0.04 to 0.07)	2%	1163	0.77	0.05 (− 0.04 to 0.14)	6%	277
Satisfied with reward received from work	0.51	0.02 (− 0.07 to 0.1)	3%	1068	0.43	0.1 (− 0.07 to 0.28)	24%	250
Satisfied with ability to use skills	0.75	− 0.03 (− 0.09 to 0.03)	− 4%	1147	0.69	0.05 (− 0.08 to 0.18)	8%	275
Satisfied with training opportunities	0.56	− 0.01 (− 0.11 to 0.08)	− 2%	1003	0.64	− 0.04 (− 0.19 to 0.1)	− 7%	251
Satisfied with security at the facility	0.86	0.02 (− 0.04 to 0.08)	3%	1080	0.81	0.02 (− 0.08 to 0.11)	2%	265
Satisfied with work conditions	0.55	− 0.03 (− 0.12 to 0.06)	− 5%	1169	0.45	0.03 (− 0.12 to 0.17)	6%	278
Satisfied with leave	0.66	− 0.04 (− 0.12 to 0.05)	− 5%	1015	0.79	− 0.07 (− 0.17 to 0.02)	− 9%	267
Satisfied with work hours	0.72	0.02 (− 0.08 to 0.11)	2%	1166	0.70	0 (− 0.12 to 0.12)	0%	279
Satisfied with teamwork	0.92	− 0.01 (− 0.05 to 0.03)	− 1%	1172	0.89	0.02 (− 0.06 to 0.09)	2%	276
Satisfied with relationship with facility management	0.86	− 0.03 (− 0.09 to 0.02)	− 4%	1138	0.87	− 0.01 (− 0.09 to 0.06)	− 2%	263
Satisfied with income overall	0.26	0 (− 0.09 to 0.1)	0%	1136	0.19	0.04 (− 0.09 to 0.17)	22%	271
Satisfied with potential for promotion	0.57	0 (− 0.09 to 0.09)	0%	1066	0.57	0.04 (− 0.09 to 0.18)	7%	256
**Average impact**^**a**^	0.75	**0 (**− **0.02 to 0.01)**	0%		0.72	**0.02 (**− **0.01 to 0.05)**	3%	

Notes: analysis of data from the health facility assessments. The impact coefficient is estimated with multivariate regression models in which the outcome is regressed on the PBF treatment, controlling for randomization block (province). Standard errors are clustered at the health zone level. For the indicators in panel b, on technical process quality, the regression models include controls for facility, patient, and health worker characteristics. More information on the regression models is presented in Additional file 1

^a^Average impacts are based on random effects meta-analysis pooling all the indicators included in each panel. The weight assigned to each indicator is presented in Additional file 1

^b^The “% change” equals the estimated PBF impact divided by the mean in control group. For the meta-analysis coefficient, the mean in control group is computed by weighing each individual indicator with the weights generated by the meta-analysis regression

**Table 2:** Effects of the PBF program on coverage of incentivized services

	**Mean in control group**	**Impact and 95% CI**	**% Change**^**b**^	**N**
Early antenatal care initiation (first trimester)	0.18	0.08 (0.03–0.13)	43%	4135
At least 4 antenatal care visits during the last pregnancy	0.34	0.03 (− 0.06 to 0.12)	9%	4134
Antenatal care with tetanus shot	0.78	0.03 (− 0.02 to 0.09)	4%	4135
Antenatal care with anti-malarial	0.71	0.01 (− 0.07 to 0.09)	1%	4135
Institutional delivery	0.91	0.03 (− 0.02 to 0.07)	3%	4089
Any postnatal care	0.39	0.03 (− 0.06 to 0.12)	8%	4135
Modern family planning method among women aged 15–49	0.05	0.03 (0.01–0.06)	67%	9585
Growth monitoring in the past 6 months for children under 5	0.03	0.01 (− 0.02 to 0.04)	35%	7247
Children aged 13–24 months with all basic vaccinations	0.51	0 (− 0.12 to 0.11)	0%	1540
**Average impact**^**a**^	0.25	**0.03 (0.02**–**0.04)**	12%	

Notes: analysis of data from the household surveys. The impact coefficient is estimated with multivariate regression models in which service utilization is regressed on the PBF treatment, controlling for randomization block (province). Standard errors are clustered at the health zone level. The sample for maternal health care indicators is of women 15–49 with a live birth in the 2 years preceding the survey

^a^Average impacts are based on random effects meta-analysis pooling all the indicators included in each panel. The weight assigned to each indicator is presented in Additional file 1

^b^The “% change” equals the estimated PBF impact divided by the mean in the control group. For the meta-analysis coefficient, the mean in the control group is computed by weighing each individual indicator with the weights generated by the meta-analysis regression

On average, the PBF program improved structural quality by 4 percentage points (ppts) (0.01–0.08), corresponding to an 8% increase (Table 1). This effect was mostly driven by changes in health centers, which saw a 4 ppts (0–0.07) increase in the availability of basic equipment and a 19 ppts (0.11–0.27) increase in the availability of family planning products. There was no improvement in the structural quality of hospitals, which had substantially higher structural quality in comparison with the health centers even in the absence of PBF.

The program increased technical process quality by 5 ppts (0.03–0.07), a relative increase of 9%. Although positive effects were estimated for both hospitals and health centers, there was some heterogeneity with respect to changes in the quality of specific services. PBF led to a major improvement in the quality of family planning consultations in health centers while, in hospitals, the largest impacts were found for postpartum care, delivery care, antenatal care, and child curative care though not all results were statistically significant at the 5% level. Non-technical process quality improved by 2 ppts (0–0.04, 2% increase). This increase was mostly driven by improvements in respectful care during delivery, child curative care, and family planning consultations recorded by clinical observers. Such differences were not detected in users’ satisfaction.

Fee policies and user satisfaction with fees improved by 14 ppts (0.07–0.20), a 20% increase relative to the unconditional financing group. Health centers and hospitals were significantly more likely to publicly post their fee schedules, and health centers were significantly more likely to charge flat fees and introduce fee exemptions for impoverished users. In addition, users expressed greater satisfaction with service affordability during exit interviews. No impact was found on facilities’ official fee schedules or fees reported to have been paid during the exit interviews. In health centers, fees for antenatal care were reduced, and expenditure reports for child curative care were lower. We did not find such effects in hospitals or in the pooled analysis.

The PBF program improved facility management scores by 9 ppts (0.04–0.15, 16% increase). Health centers were 11 ppts (0.03–0.09) more likely to have clinical protocols and were 6 ppts (0.00–0.12) more likely to duly complete reporting duties such as filling out integrated disease surveillance reports, different registries, and monthly activities reports. Both health centers and hospitals were more likely to post-infection protection and control procedures on their walls. No differences were found in health provider satisfaction.

At the population (household) level, PBF increased coverage of incentivized services by an average of 3 ppts (0.02–0.04), which corresponds to a 12% increase relative to the unconditional financing group. This increase was largely driven by increased use of modern methods of family planning by 4 ppts (0.01–0.06) and increase in the share of women who initiate antenatal care during the first trimester of their pregnancy (8 ppts (0.04–0.13)).

## Discussion

In this paper, we present the results of a first attempt to comprehensively assess the health systems impact of PBF. In comparison with unconditional transfers of equivalent size to the reward payments made through PBF, we found that PBF had positive impacts on multiple domains of health systems. At the population level, we saw small positive impact on service coverage. At the facility level, improvements were seen in structural quality, technical and non-technical process quality, facility management, and fee policies, but found no impacts on reported service fees. The PBF program also did not affect providers’ satisfaction, which could have either increased or decreased theoretically. Most of facility-level improvements were seen in both health center and hospitals. Structural quality improved only in health centers, where baseline levels were substantially lower than in hospitals.

The positive impacts on multiple domains suggest that PBF programs can achieve some of their health systems reform objectives. Considering the WHO Health Systems Framework [24], the DRC PBF program improved performance related to several of the system building blocks including service delivery, medical products, information, health care financing, and governance. On the other hand, the results also show the limitations of the PBF program. Although some positive changes were achieved, very large gaps remain with respect to service coverage and quality after 5 years of implementation. While users reported higher satisfaction with service affordability and fee policies appear to improve, we did not detect significant reductions in out-of-pocket expenditure on services. Despite the positive impacts of the program, broader reforms are required to achieve universal health coverage and substantial reductions in mortality in DRC. Larger impacts could likely be achieved with modifications of the PBF program, but additional policy changes and resources are likely also required.

Our findings differ from those of a recent pooled analysis of data from Cameroon, Nigeria, Rwanda, Zambia, and Zimbabwe that concluded that PBF offers similar gains to direct facility financing approaches that provide unconditional transfers [25]. As found in most evaluations of PBF programs in other countries, the program in DRC significantly increased coverage of only a few of the incentivized services although the types of services impacted in DRC are different. With respect to quality of care, we estimated greater improvements than the previous studies. A comparison of particular interest is with an evaluation of a previous PBF pilot in DRC that concluded that the PBF approach reduced utilization of services and providers’ satisfaction in comparison to unconditional payments to facilities [14]. The differences in the results highlight the importance of the details of the PBF design in addition to the context in which it is implemented. The two PBF models are different in several important ways such as the volume of performance payments, whether quality was incentivized, and the introduction of management tools.

This study was not designed to evaluate the effects of increased facility financing by itself, regardless of the conditionality of performance. For many outcome measures, the improvements between the baseline survey and the follow-up in the control group are bigger than the estimated differences between the PBF and control groups at follow-up [26]. While we cannot assess the exact impact the additional financing had on these outcomes rather than other factors, evidence from other countries suggests that such unconditional financing can improve some outcomes [10, 13, 15]. Facility financing, whether conditional or not, is likely to be of particular importance in a context such as DRC, where health facilities heavily rely on user fees while a large share of the population lives in extreme poverty.

This study has some important limitations. First, it focuses only on outcomes at the facility and household level and does not assess impacts of the PBF program on higher-level health system outcomes such as improvements in data systems, or changes in public financing management [27]. Second, the study may overestimate the quality of health services as health workers are likely to perform better while being observed by the research teams [28]. Our analysis relies on the assumption that the magnitude of the Hawthorne Effect is independent of the PBF treatment status given that the collected data would not affect performance payments. Finally, findings might have been affected by the COVID-19 pandemic. While disruptions to essential maternal and child health services have been documented in DRC [29], they were mostly concentrated in the beginning of the pandemic and in major urban areas that are outside of this study’s geographical coverage. The PBF program was not suspended and payments to both PBF and control facilities were not disrupted. However, if the pandemic weakened implementation of any component, or restricted facilities’ scope to increase utilization or improve their infrastructure, this might reduce the estimated impacts.

## Conclusions

The large-scale experimental evaluation of a PBF program in the DRC presented here suggests that a well-designed and implemented PBF program can lead to substantial health systems improvements compared to simple unconditional cash transfers to facilities of similar magnitude. At the same time, large gaps remain in several domains, suggesting that in a context such as the DRC, a PBF program alone is not sufficient to generate the health system improvements necessary to achieve the ambitious universal health coverage and mortality reduction targets set in the Sustainable Development Goals. There are known challenges that the PBF program was not designed to address and that should be considered by future reform efforts, such as the urgent need to increase overall financing to the under-resourced health system, the need to improve supply chains, and the need to reform the management of human resources for health. At the global level, there is a need to better understand what drives the heterogeneity in PBF impacts across settings as well as to identify the enabling conditions and primary PBF design features needed for achieving impacts.

### Supplementary Information

**Additional file 1: **Information on the DRC performance-based financing program, the randomization, the data collection, the definition of the outcome measures, the statistical methods, and provides additional results. **Table S1. **PBF quantity indicators and corresponding relative weights - Minimum Package of Activities (Health centers). **Table S2. **PBF quantity indicators and corresponding relative weights - Minimum Package of Activities (Hospitals). **Table S3. **Quality checklist components and weights for the minimum package of activities (health centers). **Table S4. **Quality checklist components and weights for the complementary package of activities (hospitals). **Figure S1. **Spatial location of study areas and results of the intervention randomization. **Table S5. **Baseline balance of outcomes measured through health facility assessments. **Table S6.** Baseline balance of outcomes measured through household surveys. **Table S7.** Q-values and meta-analysis weights for outcomes at the health facility level. **Table S8.** Q-values and meta-analysis weights for incentivized services. **Figure S2.** Pooled analysis of structural quality outcomes. **Figure S3.** Pooled analysis of technical process quality outcomes. **Figure S4.** Pooled analysis of non-technical quality outcomes. **Figure S5.** Pooled analysis of service fees outcomes. **Figure S6.** Pooled analysis of fee policies and user satisfaction with affordability outcomes **Figure S7.** Pooled analysis of facility management outcomes. **Figure S8.** Pooled analysis of providers’ satisfaction outcomes.

## Data Availability

The data used for this study is available upon request through the World Bank Microdata Library.

## Abbreviations

DRC: Democratic Republic of Congo
PBF: Performance-based financing
WHO: World Health Organization
