# Supplementary material for: Impacts of performance-based financing on health system performance: evidence from the Democratic Republic of Congo
Source: BMC Med. 2023 Oct 4;21:381. doi: 10.1186/s12916-023-03062-8 (PMC10552286; doi:10.1186/s12916-023-03062-8)

# Supplementary Information

## The Performance-Based Financing Program

The Performance-Based Financing intervention studied in this paper was introduced in the context of the Health System Strengthening for Better Maternal and Child Health Results Project (*PDSS – Le Projet de Développement du Système de Santé*) that was financed by the World Bank since 2015. For additional information on the project and the PBF program, please refer to the impact evaluation report of the project (Shapira et al. 2022), from which some of the text in this appendix is adapted.

The financing received through the intervention provided additional funds to facilities, without replacing any previously received sources of funding. There were two types of performance contracts health facilities signed with provincial purchasing agencies. Independent and not-for-profit entities were created for the management of the contracts and verification of performance. Health centers were contracted to deliver the Minimum Package of Activities (MPA), a set of core preventive and curative primary health services. General referral hospitals and some referral health centers were contracted to deliver the Complementary Package of Activities (CPA), including services to be delivered at first level referral such as complicated deliveries, blood transfusions, and surgeries requiring anesthesia. The facility performance payments were transferred directly to facility bank accounts.

The PBF program introduced rules for how contracted health facilities could spend their revenues, whether received as PBF payments or through any other source. The spending by each facility needed to be aligned with a quarterly business plan in which facilities documented their performance on the different indicators in the previous quarter, set targets for the new quarter, and defined strategies for achieving these targets. Facilities were allowed to spend a maximum of 50% of each quarter’s revenue on personal bonuses for staff. From 2020 on, facilities were also required to spend a minimum of 20% of the PBF payments on medications and other consumables. The rest of budget needed to cover all operational costs, investments in facility infrastructure and equipment, and any savings.

The program also dictated how individual performance bonuses were distributed among the facility’s staff. Thirty percent of the staff bonus (or up to 15% of the overall quarterly facility revenue) was fixed in the sense that it did not depend on individual staff performance. The relative share each staff member received was determined according to their title (e.g. medical doctor, nurse of a specific grade), responsibility within the facility (e.g. head of maternity, facility in-charge), and seniority. The other 70% of the staff bonus was distributed based on an individual performance evaluation of each staff member. Staff performance was scored by the facility-in-charge with a use of a performance checklist developed by the program. Once all the staff members were evaluated, the relative individual scores determined the share everyone received.

To account for the challenging geographical context with lack of a road network in most areas, and the unequal distribution of the population and health providers, the performance payments paid to facilities were adjusted using so-called “equity classifications”. Base performance fees were adjusted by up to 80% according to classification of both health zones and health facilities. The PBF intervention also aimed to address equity concerns at the local level by providing facilities higher financial incentives for treating the poorest in each health area. The poorest users were identified by a community targeting exercise led by dedicated committees that were formed in the catchment area of contracted facilities. In each health area, up to 5% of households could be identified as most impoverished, who received free services while facilities receive higher performance payments for treating them.

Quantity indicators (incentivized services)

Provision of preventive and curative services was incentivized on a fee-for-service basis, with fees set prospectively. The specific services and their relative weights are presented in Tables A2 and A2. The rewards are presented in terms of weights because the actual fee depended on the equity classification of each facility. Some changes to the list of services and to the relative weights were introduced after the project’s midterm review in 2019.

Table S1: PBF quantity indicators and corresponding relative weights - Minimum Package of Activities (Health centers)

| **Category** | **Service** | **Relative weight before midterm review (2016-2019)** | **Relative weight After midterm review (since 2019)** |
| --- | --- | --- | --- |
| **Outpatient consultation** | New case | 1 | 1 |
|  | New case for indigent patient | 3 | 4 |
| **Surgery** | Minor surgery (without need for anesthesia) | 5 | 3 |
| **Referral** | Referral of a severe case to a higher-level facility | 15 | 9.2 |
| **Child vaccination** | Child completely vaccinated in first year of life | 25 | 8.3 |
| **Antenatal care** | Two or more Tetanus Toxoid injections during pregnancy | 3 | 2 |
|  | First consultation in first three months of pregnancy | 2 | 2 |
|  | Fourth Antenatal care visit |  | 1.7 |
|  | Third dose of intermittent preventive treatment (Sulfadoxine/Pyrimethamine) |  | 2 |
| **Delivery** | Delivery with skilled birth-attendant with a filled partograph | 17 | 9.2 |
| **Postnatal care** | Consultation between 3 and 7 days after delivery | 4 | 2 |
| **Family planning** | New user of pills or injectables | 13 | 5 |
|  | New user of IUD or implants | 15 | 5 |
| **Growth monitoring** | Child 6-23 months | 0.8 | 1 |
|  | Child 24-59 months | 0.4 | 0.7 |
| **Home visit** |  | 2.5 | 0.8 |
| **HIV** | Voluntary counselling and testing | 3 | 1 |
|  | PMTCT | 20 | 10 |
|  | Care for newborn of HIV+ woman | 25 | 13.3 |
|  | Biannual follow-up with individuals on antiretroviral regime |  | 20 |
| **Tuberculosis** | Detection of positive case | 75 | 36.7 |
|  | Case treated and recovered | 150 | 75 |

Note: The rewards for the quantity indicators are presented in terms of weights because facilities received different dollar amounts based on their equity classification. For benchmarking, the base performance fee for outpatient consultation was $0.6 USD after the midterm review, and the base fee for an outpatient consultation for an indigent patent was $2.4 USD. For facilities in the highest equity classification, the corresponding fees were $1.1 and $4.4.

Table S2: PBF quantity indicators and corresponding relative weights – Complementary Package of Activities (Hospitals)

| **Category** | **Indicator** | **Relative weight before midterm review (2016-2019)** | **Relative weight After midterm review (since 2019)** |
| --- | --- | --- | --- |
| **Outpatient consultation** | Outpatient care for a referred patient | 5 | 10 |
| **Inpatient care** | Day of hospitalization | 1 | 1 |
|  | Day of hospitalization for indigent patient | 3 | 3 |
| **Surgery** | Minor surgery | 6 | 5.8 |
|  | Minor surgery for indigent patient | 10 | 10 |
|  | Major surgery | 25 | 50 |
|  | Major surgery for indigent patient | 75 | 75 |
| **Blood transfusion** | Blood transfusion | 6 | 5.8 |
| **Antenatal care** | First visit in first three months of pregnancy | 2 | 3 |
|  | Fourth Antenatal care visit |  | 2 |
|  | Third dose of intermittent preventive treatment (Sulfadoxine/Pyrimethamine) |  | 2 |
| **Delivery** | Uncomplicated delivery | 15 | 15 |
|  | Caesarian delivery | 70 | 70 |
|  | Complicated delivery (Excluding Caesarian) | 30 | 30 |
| **Family planning** | New user of pills or injectables | 8 | 8,3 |
|  | New user of IUD or implants | 15 | 15 |
|  | Sterilization (Masculine or feminine) | 50 | 50 |
| **HIV** | Voluntary counselling and testing | 2 | 1.7 |
|  | PMTCT | 15 | 15 |
|  | Care for newborn of HIV+ woman | 20 | 20 |
|  | Initiation of antriretroviral treatment | 15 | 15 |
|  | Antiretroviral client follow-up | 10 | 25 |
| **Tuberculosis** | Detection of positive case | 75 | 75.8 |
|  | Case treated and recovered | 150 | 150.8 |

Note: The rewards for the quantity indicators are presented in terms of weights because facilities received different dollar amounts based on their equity classification. For benchmarking, the base performance fee for inpatient care at the hospitals was $0.6 USD per day after the midterm review, and the base fee for such care for an indigent patent was $1.8 USD. For hospitals in the highest equity classification (category 5), the corresponding fees were $0.8 and $2.4.

Quality indicators and quality bonus

The quality checklists used to determine facilities’ overall quality score contained a range of indicators divided into sub-categories. Tables A3 and A4 present the different categories, examples of indicators in each category, and the weight given to each category in the overall score for facilities contracted to provide the MPA and CPA before and after the changes introduced during the project’s midterm review.

Table S3: Quality checklist components and weights for the minimum package of activities (health centers)

| **Quality Category** | **Share of overall score before midterm review** | **Share of overall score after midterm review** |
| --- | --- | --- |
| 1. General organization   e.g. presence, completion and archival of different forms and reports, catchment area map, means of communication | 8% | 8% |
| 1. Management plan   Quarterly management plan completed with different elements such as outreach strategies and negotiated fees | 2% | 6% |
| 1. Financial management   e.g. financial and accounting documents filled and present, compensation structure clear to providers | 4% | 3% |
| 1. Indigent committee   e.g. reports of monthly indigent committee meetings, documentation of care provided to indigent patients | 5% | 4% |
| 1. Hygiene and sterilization   e.g. waste disposal, sterilization procedure and supplies, containers for sharps and needles, cleanliness | 8% | 6% |
| 1. Outpatient consultations   e.g. management of registries and forms, physical state of waiting and consultation rooms, review of registers for child curative care, availability of supplies | 28% | 21% |
| 1. Family planning   e.g. staff member trained, confidentiality at consultation room, availability of family planning products | 9% | 8% |
| 1. Laboratory   e.g. presence of lab technician, management of registries, availability of equipment and supplies | 5% | 6% |
| 1. Inpatient care   e.g. management of registries, condition of observation areas (cleanliness, lighting, beds) | 2% | 4% |
| 1. Medications and consumables   e.g. management of stock inventories, correct stocking of products, correct disposal of drugs | 5% | 5% |
| 1. Tracer drugs   Availability of pre-specified list of essential drugs and consumables | 6% | 3% |
| 1. Maternity   e.g. correct use of partograms, availability of equipment and supplies, cleanliness and confidentiality in delivery room | 7% | 12% |
| 1. Vaccination   e.g. availability of vaccines, storage of vaccines, management of registries and forms | 5% | 7% |
| 1. Antenatal care   e.g. availability of equipment and supplies, review of registries | 3% | 3% |
| 1. HIV/TB   e.g. confidential consultation room, trained staff members, review of registries | 2% | 5% |

Table S4: Quality checklist components and weights for the complementary package of activities (hospitals)

| **Quality Category** | **Share of overall score before midterm review** | **Share of overall score after midterm review** |
| --- | --- | --- |
| 1. General organization | 4% | 6% |
| 1. Management plan | 1% | 3% |
| 1. Financial management | 7% | 4% |
| 1. Indigent committee | 3% | 3% |
| 1. Hygiene and sterilization | 7% | 9% |
| 1. Outpatient consultations | 17% | 17% |
| 1. Family planning | 5% | 4% |
| 1. Laboratory | 2% | 4% |
| 1. Inpatient care   e.g. management of registries, condition of observation areas (cleanliness, lighting, beds) | 25% | 16% |
| 1. Medications and consumables | 3% | 3% |
| 1. Tracer drugs | 5% | 3% |
| 1. Maternity | 6% | 7% |
| 1. Antenatal care | 1% | 5% |
| 1. HIV/TB | 1% | 4% |
| 1. Surgery | 13% | 12% |

The quality bonus was determined proportional to the *quantity* payment. Facilities received no quality bonus if they scored less than 50% on the quality checklist. Health centers could receive a maximum bonus of 25 percent of the quantity payment, while hospitals could get a maximum bonus of 40% of the total quantity-based transfers made. The overall quarterly payment can be represented as follows:

$$\text{Quarterly Payment for facility j}=\left\{ \begin{aligned} e_{j}\sum_{i} s_{ij}f_{ij} \text{ ,if }Quality\_Score<50\% \\ e_{j}\left( \sum_{i} s_{ij}f_{ij} \right)\left( 1+\gamma_{j}{Quality\_Score}_{j} \right)\text{ ,if }Quality\_Score>50\% \end{aligned} \right.$$

where $s_{ij}$ represents the quantity of incentivized service $i$ that facility $j$ provided during the quarter and $f_{ij}$ represents the fee-per-service associated with the service, depending on the package of incentivized services. $e$ represents the equity adjustment and ranges between 1 and 1.8. That is, $e_{j}\sum_{i} s_{ij}f_{ij}$is the *quantity payment*. ${Quality\_Score}_{j}$ is the quarterly quality score in a range between 0 and 100 percent. $\gamma_{j}$ equals 0.25 for health centers and 0.4 for hospitals.

Verification and counter-verification

Quantity indicators were reported by each contracted health facility and verified by the provincial purchasing agencies by reviewing health management information system reports and facility registries. Then, a random sample of 3 percent of patients (but no more than 50 per facility per quarter) were drawn from the facility registries for community verification by contracted community associations in each health zone. Health zone management teams and the Provincial Health Divisions determined the quality scores through completion of the quality checklists. The health zone teams manage this process for health centers, whereas the provincial teams do this for hospitals. Health zone management teams, provincial teams, and the provincial purchasing agencies are all under performance contracts with payments conditional on timely completion of their corresponding tasks.

A third-party counter-verification agency validated the verified performance in a sample of facilities, chosen both randomly and based on large disparities between claimed and verified data. Penalties were applied in instances of disparities and contract suspension applied when disparities were found more than twice.

**Reference**

Shapira, Gil; Samaha, Hadia; Fritsche, Gyorgy Bela; Lushimba, Michel Muvudi; Guenther Fink.2022. Performance-based Financing in the Health Sector of the Democratic Republic of Congo: Impact Evaluation Report. Washington, D.C.

## Randomization

Randomization of the PBF program at the health zone level was blocked by province. In Kwilu province, which includes the largest number of study health zones (22), health zones were split into 2 remoteness classes and randomization was blocked also by these classes. For transparency about the program allocation, randomization was conducted during provincial public randomization ceremonies in the presence of representatives from all health zones. A transparent bucket contained folded notes with indicating participation in the PBF program or in the control group. Health zone representatives drew the folded notes. The results of the randomization were written in a dedicated form that was signed by participants from the program management team, provincial health teams, and civil society representatives. The map in Figure A1 displays the results of the randomization. Balance testing with respect to baseline sample characteristics and outcomes are presented in Appendix 5.

**Figure S1**: **Spatial location of study areas and results of the intervention randomization**


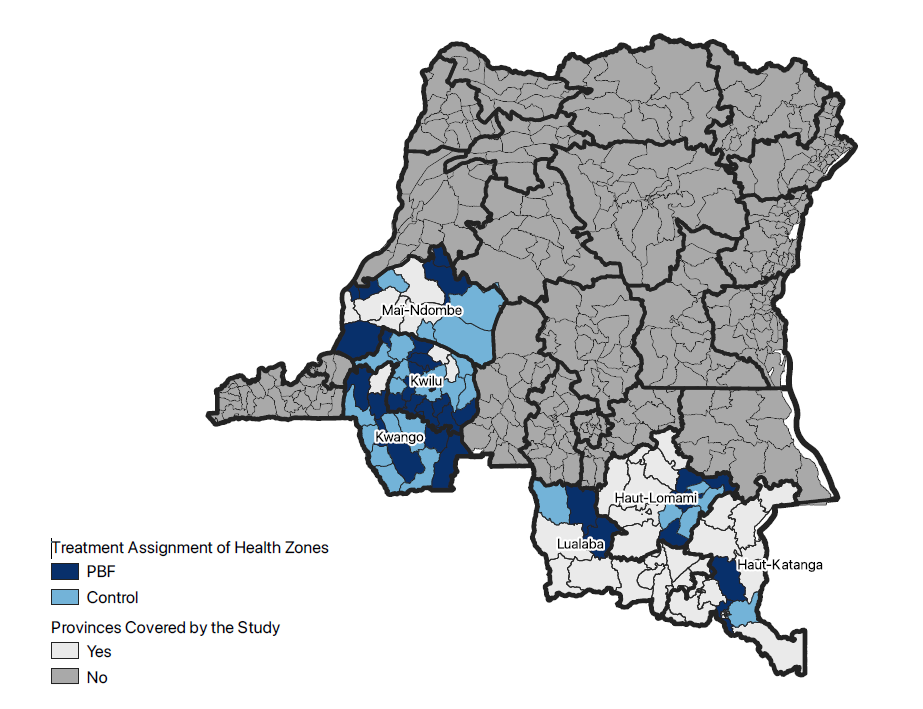


## Information on data collection

The survey instruments were adapted to the country context from those developed for the Impact Evaluation Toolkit of the Health Results Innovation Trust Fund ([www.rbfhealth.org/resource/impact-evaluation-toolkit-provides-hands-guidance](http://www.rbfhealth.org/resource/impact-evaluation-toolkit-provides-hands-guidance)). The adaptation process was led by the research team and the organization Medecins d’Afrique, that was contracted to implement the survey, and included consultations with the Ministry of Public Health. The survey tools and data are available in the World Bank Microdata Library ([microdatalib.worldbank.org](https://microdatalib.worldbank.org/index.php/home)).

The household and facility-based surveys were conducted by different dedicated teams. Given the strong focus on reproductive and maternal health, all enumerators for the household survey were female. All team members conducting the facility-based surveys had prior medical training. Observations of antenatal care and family planning consultations and of deliveries were only conducted by female enumerators.

### Household Survey

The household survey was composed of two main sections. For the first section, the household head or most knowledgeable household member available at the day of the survey were interviewed about household-level characteristics, such as household composition, and economic situation of the household (dwelling conditions, asset ownership, etc.). For the second component, women 15 to 49 years old were interviewed about their health and that of their children under the age of 5. The main themes of the women interviews were: general health and case management of illness, fertility and family planning, care provided during recent pregnancies, immunization and vaccination of children under 5.

### Facility-based survey

The facility-based survey was composed of the following modules:

- General facility assessment: The main respondent for the general facility assessment module was the facility manager or the person in charge of the health facility at the time of the visit b the survey team. The themes covered by the assessment include facility staffing, financing and user fees, infrastructure, availability of equipment, drugs and other medical supplies, and record keeping and reporting.
- Health provider interview: Health workers with reproductive, maternal and child health service delivery responsibilities we interviewed. The survey teams were instructed to prioritize interviewing providers who were observed during the direct clinical observations described below. Otherwise, providers present at the facility were selected randomly. The interview covered themes such as roles and responsibilities, training, satisfaction, and compensation and benefits.
- Direct clinical observations: technical process quality was measured with the use of direct clinical observations. Enumerators used structured observation tools to record content of services. Observations of curative consultations for children under 5 and antenatal consultations were conducted during the baseline and follow up data collection. Observations of family planning consultations and deliveries were conducted only during the follow up. Due to need for additional training and time spent in facilities, observations of labor and delivery services were only conducted in the provinces of Kwango, Kwilu and Mai Ndombe.
- Patient exit interviews: interviews were conducted with users whose antenatal and family planning consultations were observed and the guardians of the children whose curative care consultations were observed. The interviews were used to collect background information on the patients and assess the patients’ satisfaction with the care received.

## Definition of outcome measures

Structural quality

All outcome measures in the structural quality domain are indexes defined as the average availability of different items confirmed through direct observations during the facility assessments. Equal weights are provided to each item in the creation of each index. The items included in the construction of each index are listed below:

1. Basic equipment index: thermometer, adult stethoscope, child stethoscope, blood pressure cuff for adult, blood pressure cuff for child, otoscope, delivery room light, partograph, measuring tape, aspirator, delivery bed, obstetric stethoscope, absorbable suture, non-absorbable suture, sterile gauze, clamp, baby scale, adult scale, microscope, centrifuge, needle holder, protective apron, gloves, delivery kit/box, autoclave, electric sterilizer, steam sterilizer.
2. Essential medicine and consumables index: paracetamol, ibuprofen, chlorpheniramine, oxytocin, mebendazole, iron sulfate, amoxicillin pill, amoxicillin injection, amoxicillin syrup, cotrimoxazole pill, cotrimoxazole syrup, doxycycline, sulfadoxine/pyrimethamine, oral rehydration salts, metronidazole, gloves, catheter (18g and 22g), infuser, ringer solution, isotonic glucose, needle
3. Vaccines index: BCG, pentavalent, polio, yellow fever, measles, tetanus toxoid.
4. Family planning products index: contraceptive pill, male condom, female condom, diaphragm, morning after pill, injectable contraceptive, implant, intrauterine device
5. Infrastructure index: fencing of facility area, laboratory, toilets, phone, vehicle for patient transfer, electricity, dedicated and secured area for drug storage
6. Infection prevention and control index: functioning incinerator, improved source of water, container for sharps and needles, handwashing stations in consultation rooms

Technical process quality

All outcomes in the technical process quality domain are constructed from data collected through direct clinical observations of consultations and deliveries. Observations of antenatal care are restricted to first antenatal consultations women had during their pregnancies. Observations of child curative care (IMCI) are restricted to children under-5 who are seen by a health worker in the first time for their illness.

1. Antenatal care score: an unweighted average of completion of the following 28 elements in 5 domains:
   1. Medical history-taking: ask about age, medications, last cycle, previous pregnancies (bleeding, stillbirths, abortion, assisted deliveries), bleeding during current pregnancies, fever, headache or blurry vision, swelling, fatigue
   2. Physical exams: provider washes hands before touching pregnant woman, measure blood pressure, weight, check swelling, palpate abdomen
   3. Diagnostic tests: HIV, syphilis, hemoglobin level, urine test
   4. Preventive care: iron with or without folic acid, tetanus toxoid shot, malaria chemoprophylaxis
   5. Counselling topics: nutrition during pregnancy, birth preparation, pregnancy danger signs
2. IMCI: assessment score – an unweighted average of completion of the following 21 elements: greet patient, weigh child, ask about diarrhea, skinfold check, ask about cough, ask about stridor, check respiratory frequency, undress child, auscultation, ask about fever, asks about measles, check eyes, check nose, check skin, check throat, check ears, check neck, ask about ear pain, check palms, check feet, test for malaria
3. IMCI: diagnosis score – binary indicator: the health provider gave the right diagnosis for malaria, severe pneumonia, or severe dehydration based on the symptoms, test results, and the IMCI guidelines
4. IMCI: correct treatment – binary indicator: correct provision of antimalarials, antibiotics and fluids based on IMCI guidelines, child symptoms and test results.
5. IMCI: no unnecessary treatment –binary indicator: the health provider does not prescribe antimalarials or antibiotics when not necessary according to the IMCI guidelines.
6. Family planning consultation score: an unweighted average of completion of the following 17 elements, informed by Jain et al (2019):
   1. Method selection: ask if client want any more pregnancies in the future, ask about preference for a method, ask about past contraceptive use, discuss multiple methods, doesn’t show bias towards one method
   2. Effective use: explain how to use method, explain how the method works, explain side effects, explain how to manage side effects
   3. Continuity of use: discuss when the method needs to be renewed, where to renew or restock, possibility of changing methods, suggest a follow up consultation
7. Delivery score - an unweighted average of completion of the following 21 elements, informed by Jain et al (2019): provider explain sequence and objective of each procedure, ask about HIV status, ask about blurred vision during pregnancy, ask about bleeding during pregnancy, measure blood pressure, wash hand before physical exams, wear gloves for vaginal exam, disinfect hands before vaginal exam, use partograph, neonatal resuscitator laid out in delivery room, uterotonic injection, assess completeness of placenta and membranes, assess perineal and vaginal lacerations, keep baby warm, place newborn on mother’s abdomen, cut umbilical cord, check for bleeding, blood pressure and pulse and palpate uterus in first hour after delivery, assist mother in initiating breastfeeding
8. Postpartum care score - an unweighted average of completion of the following 4 elements, informed by WHO (2015): assess bleeding, blood pressure and pulse and check uterus at least four times in the hour after birth and at least once in the following hour, assess bleeding in the third stage of delivery
9. Newborn care score: an unweighted average of completion of the following 8 elements, informed WHO (2015; 2017): keep newborn warm, place newborn on mother’s abdomen for skin-to-skin, cut umbilical cord, assist mother in initiating breastfeeding, take APGAR scores, measure newborn temperature, weigh newborn, clean newborn eyes

Non-technical process quality

Two types of data sources are used for the non-technical process quality measures: users’ satisfaction elicited during exit interviews and observations of enumerators conducting the direct clinical observations.

During exit interviews, respondents were asked whether they are satisfied with different aspects of the facility and the consultation. The measures of user satisfaction are constructed with unweighted averages over the following seven aspects:

- Facility cleanliness
- Respect by health providers
- Providers’ explanations
- Waiting time
- Confidentiality
- Duration of consultation
- Hours of operation

The respect index recorded by enumerators for each service is constructed as an unweighted average of the following elements:

1. ANC: provider explained procedures, provider encouraged pregnant woman to ask questions, provider respected confidentiality
2. Child curative care: provider greeted patient and guardian, provided explained procedures, provider respected confidentiality, providers’ behavior was appropriate
3. Family planning consultations: provider greeted user, provider addressed the user respectfully, provider encouraged questions, provider respected user’s privacy
4. Delivery care: provider explained procedures, no physical abuse during delivery, no verbal abuse during delivery, provider protected confidentiality

Service fees

Two data sources are used as service fee measures. The first are official fees reported by facility managers during the general facility assessments. The second source is the exit interviews, during which users reported how much they had paid for ANC, child curative care, and family planning consultations.

Fee policies and user satisfaction with fees

The following measures have been extracted during the interviews with facility managers:

1. Flat fees: binary variable - whether the facility charges flat fees (and does not charge separately for consultations, laboratory testing, etc.)
2. Fee posted: binary variable - whether facilities post user fees in a space open to the public (verified through direct observation by enumerator)
3. Fee exemptions for poor users: binary variable - whether the facility provide at least some fee exemptions for the poorest users in their catchment area.

During the exit interviews for ANC, child curative care and family planning consultations, respondents reported whether they agree that service fees were affordable. The outcome is captured by a binary variable.

Facility management

1. Protocols index: an unweighted average of the availability of protocols and directives related to sterilization, malaria, acute respiratory infection, diarrhea, treatment directives, tuberculosis, health management information system, child vaccination, reproductive health, sexually transmitted infections, HIV, PMTCT, essential drugs, drug supplies, drug side effects, EMONC.
2. Reporting index: enumerators verified whether reports and registries are dully filled. The index is constructed with unweighted averages over the following items: monthly activity reports, monthly vaccination report, integrated disease surveillance report, monthly HIV/AIDS report, PMTCT report, family planning registry, antenatal care registry, delivery registry
3. Posting of infection and control procedures index: an unweighted average over whether facility has sterilization and disinfection procedures posted on the walls.
4. Mechanism to seek patients’ opinions: binary variable reported by facility manager.

Providers’ satisfaction

Health workers’ satisfaction is measured during the health providers’ interviews. The respondents declared whether they are satisfied, dissatisfied or neither satisfied nor dissatisfied with respect to a series of aspects related to their work. For each aspect, a binary variable equals 1 if the respondent declared being satisfied.

**References:**

Jain, A., Aruldas, K., Mozumdar, A., Tobey, E. and Acharya, R., 2019. Validation of two quality of care measures: results from a longitudinal study of reversible contraceptive users in India. *Studies in family planning*, *50*(2), pp.179-193.

Tripathi, V., Stanton, C., Strobino, D. and Bartlett, L., 2019. Measuring the quality of maternal and care processes at the time of delivery in sub-Saharan Africa: development and validation of a short index. *BMC pregnancy and childbirth*, *19*(1), pp.1-12.

World Bank, 2014. *Revue des dépenses publiques en RDC – santé*. World Bank – unpublished report.

World Health Organization. 2015. “Pregnancy, childbirth, postpartum and newborn care: a guide for essential practice (3^rd^ edition).” <https://www.who.int/publications/i/item/pregnancy-childbirth-postpartum-and-newborn-care>.

World Health Organization, 2017. *WHO recommendations on newborn health: guidelines approved by the WHO Guidelines Review Committee* (No. WHO/MCA/17.07). World Health Organization.

## Statistical appendix

### Sampling framework

The sampling for the study relied on lists prepared by the different provincial health teams in 2014. The lists included the health centers in each health zone and the villages or urban neighborhoods in the catchment area of each health center. The research team first randomly selected five health centers within each health zones for the implementation of the facility-based surveys. Then, within the catchment area of each selected health center, a single village or neighborhood was selected for the household survey.

The original sample for the study included 100 health zones in 11 provinces. This sample represented all the zones originally supported by the World Bank-financed Health System Strengthening Project where a PBF pilot was not conducted as part of a previous project. Additional two health zones were removed from the study due to implementation of other interventions in addition to the project. In May 2018, there was an Ebola outbreak in Equateur province. As part of the project’s response to the outbreak, health zones supported by the project in the northeast of the country received additional finacngin to maintain health services and for recovery. Given this change in financing in both PBF and control health zones, and the higher-than-expected survey costs incurred during the baseline, it was decided to remove from the study the 39 health zones in the provinces or Equateur, Mongala, Sud-Ubangi, and Tshuapa. It was subsequently decided to also remove three participating health zones from Maniema province. The reduction in the number of covered health zones from 100 to 58 reduces the statistical power of the study. As PBF was allocated at the health zone level, the standard errors in all of the regressions are clustered at the health zone level and the expansion of the sample sizes in the remaining zones would have limited impact on power. Nevertheless, as the randomization was blocked by province, the removal of provinces does not impact the internal validity of the study.

To determine the required number of households to sample in each village or neighborhood in the baseline survey, data from the 2007 Demographic and Health Survey were used, which was the most recent household data available at the time. The study was powered to detect difference in percentage of children fully vaccinated. The rate of fully vaccinated children was 45% and the intra-class correlation was 0.1 at the cluster level, and 0.035 at the provincial level. It was assumed that sampling from five different villages in each health zone would bring down the intra-class correlation to 0.05. A sample of 10 household per enumeration area would enable detection of an effect of 0.075 with power 0.8 and an effect of 0.081 with power 0.9 with an alpha of 0.05.

The same health facilities and villages visited during the baseline survey were re-visited during the follow-up survey. Given the change in the number of health zones (clusters), new power calculations were conducted using the baseline survey data. The calculations assumed power 0.8 and an alpha of 0.05. A sample of 12 household per village (and 60 per health zone) would enable detection of an effect of 0.1 on the share of children 13-24 months old receiving the third dose of the pentavalent vaccine, and an effect of 0.07 on the share of recently pregnant women who initiated antenatal care in the first trimester of their pregnancy. As during the baseline survey, the household survey targeted women with recent pregnancies. In order to reflect the greater focus on family planning and adolescent reproductive health by the World Bank’s Health Nutrition and Population program in the country, additional samples were added. First, a sample of women 15-49 years old *without* a recent pregnancy was added to measure broader program impacts on use of family planning methods. Second, households with women of ages 15-20 were oversampled.

### Balance testing

To assess internal validity of the experimental design, we test for balance in outcomes between the two treatment groups at baseline. To estimate the difference between the groups, we use the same regression specification that is used for estimating the PBF impact with the follow-up data. The different outcomes are regressed on the PBF treatment, controlling for randomization block. Standard errors are clustered at the health zone level, the unit of randomization.

Table S5: Baseline balance of outcomes measured through health facility assessments

| **Variable** | **Health Centers** | | | | **Hospitals** | | | |
| --- | --- | --- | --- | --- | --- | --- | --- | --- |
|  | **Mean in control group** | **Mean in PBF group** | **Difference** | **p-value** | **Mean in control group** | **Mean in PBF group** | **Difference** | **p-value** |
| **Panel a: structural quality** | | | | | | | | |
| Basic equipment index | 0.31 | 0.31 | -0.01 | 0.661 | 0.58 | 0.60 | 0.02 | 0.547 |
| Essential medicine and consumables index | 0.44 | 0.46 | 0.02 | 0.648 | 0.69 | 0.71 | 0.04 | 0.365 |
| Vaccines index | 0.05 | 0.04 | 0.01 | 0.636 | 0.01 | 0.00 | -0.02 | 0.227 |
| Family planning products index | 0.10 | 0.15 | 0.04 | 0.191 | 0.19 | 0.31 | 0.13 | 0.161 |
| Infrastructure index* | 0.37 | 0.34 | -0.03 | 0.298 | 0.57 | 0.56 | 0.02 | 0.643 |
| Infection prevention and control index | 0.36 | 0.37 | 0.00 | 0.904 | 0.70 | 0.75 | 0.06 | 0.523 |
| **Panel b: technical process quality** | | | | | | | | |
| Antenatal care score | 0.66 | 0.71 | 0.05 | 0.102 | 0.75 | 0.81 | 0.05 | 0.335 |
| IMCI: assessment score | 0.52 | 0.49 | -0.01 | 0.817 | 0.63 | 0.60 | -0.02 | 0.631 |
| **Panel c: non-technical process quality** | | | | | | | | |
| Antenatal care respect index* | 0.81 | 0.86 | 0.01 | 0.767 | 0.84 | 0.89 | 0.03 | 0.685 |
| Antenatal care user satisfaction | 0.92 | 0.92 | 0.00 | 0.928 | 0.88 | 0.87 | 0.00 | 0.954 |
| Child curative care respect index | 0.43 | 0.44 | -0.06 | 0.396 | 0.67 | 0.59 | -0.06 | 0.415 |
| Child curative care user satisfaction | 0.88 | 0.81 | -0.04 | 0.254 | 0.84 | 0.84 | 0.01 | 0.722 |
| **Panel d: service fees (in Congolese Francs)** | | | | | | | | |
| ANC fee reported by facility | 2913 | 2115 | -770 | 0.197 | 2913 | 2115 | -770 | 0.183 |
| Delivery fee reported by facility | 12200 | 10150 | -1686 | 0.917 | 12200 | 10150 | -1686 | 0.547 |
| Curative care fee reported by facility (not specific for child care) | 2922 | 1083 | -2115 | 0.421 | 2922 | 1083 | -2115 | 0.167 |
| Child curative care fees reported in exit interviews | 8898 | 10180 | 1414 | 0.719 | 8898 | 10180 | 1414 | 0.401 |
| **Panel e: fee policies and user satisfaction with fees** | | | | | | | | |
| Flat fees | 0.51 | 0.49 | 0.01 | 0.899 | 0.20 | 0.14 | -0.05 | 0.726 |
| Fees posted | 0.60 | 0.57 | -0.02 | 0.732 | 0.53 | 0.79 | 0.25 | 0.174 |
| Fee exemptions for poor users | 0.99 | 1.00 | 0.01 | 0.311 | 1.00 | 1.00 | 0.00 |  |
| Satisfaction with service affordability in antenatal care exit interviews | 0.76 | 0.86 | 0.08 | 0.120 | 0.72 | 0.89 | 0.18 | 0.017 |
| Satisfaction with service affordability in child care exit interviews | 0.62 | 0.68 | 0.08 | 0.415 | 0.82 | 0.84 | 0.06 | 0.608 |
| **Panel f: facility management** | | | | | | | | |
| Protocols index | 0.30 | 0.30 | 0.01 | 0.776 | 0.57 | 0.61 | 0.08 | 0.412 |
| Posting of infection and control procedures index | 0.09 | 0.09 | 0.00 | 0.902 | 0.36 | 0.25 | -0.16 | 0.291 |
| Mechanism to Seek patients’ opinions | 0.46 | 0.52 | 0.04 | 0.510 | 0.40 | 0.64 | 0.30 | 0.127 |
| **Panel g: providers’ satisfaction (on a scale from 0 to 10)** | | | | | | | | |
| Satisfied with information on own performance* | 7.15 | 6.80 | -0.32 | 0.088 | 7.30 | 7.52 | 0.24 | 0.557 |
| Satisfied with level of autonomy* | 6.97 | 6.86 | -0.09 | 0.599 | 6.73 | 6.78 | 0.04 | 0.897 |
| Satisfied with the relationship with the local facility committee* | 6.04 | 6.40 | 0.34 | 0.180 | 4.57 | 6.07 | 1.12 | 0.034 |
| Satisfied with support from supervisor* | 6.74 | 6.62 | -0.13 | 0.576 | 6.80 | 6.76 | 0.00 | 0.991 |
| Satisfied with recognition received from supervisor* | 7.06 | 7.17 | 0.10 | 0.574 | 6.92 | 7.22 | 0.45 | 0.168 |
| Satisfied with reward received from work* | 5.18 | 5.29 | 0.07 | 0.739 | 4.53 | 4.80 | 0.59 | 0.235 |
| Satisfied with ability to use skills* | 6.91 | 7.09 | 0.18 | 0.304 | 7.00 | 6.89 | 0.17 | 0.619 |
| Satisfied with training opportunities* | 4.87 | 4.96 | 0.08 | 0.816 | 5.62 | 6.00 | 0.72 | 0.199 |
| Satisfied with security at the facility* | 7.35 | 7.40 | -0.01 | 0.952 | 7.03 | 7.19 | 0.07 | 0.870 |
| Satisfied with income overall* | 2.69 | 2.71 | 0.01 | 0.980 | 2.77 | 2.80 | 0.29 | 0.418 |
| Satisfied with potential for promotion* | 5.39 | 5.34 | 0.01 | 0.982 | 6.03 | 4.91 | -0.93 | 0.096 |

**Notes**: analysis of data from the health facility assessments. The difference between the two groups is estimated with multivariate regression models in which the outcome is regressed on the PBF treatment, controlling for randomization block (province). Standard errors are clustered at the health zone level. For the indicators in Panel b, on technical process quality, the regression models include controls for facility, patient and health worker characteristics. Outcome denoted with a asterisk ‘*’ are constructed differently from how they are defined for the follow up outcomes given differences in baseline survey design.

Table S6: Baseline balance of outcomes measured through household surveys

|  | **Mean in control group** | **Mean in PBF group** | **Difference** | **p-value** |
| --- | --- | --- | --- | --- |
| Early antenatal care initiation (first trimester) | 0.13 | 0.15 | 0.00 | 0.937 |
| At least 4 antenatal care visits during last pregnancy | 0.42 | 0.44 | 0.03 | 0.569 |
| Antenatal care with tetanus shot | 0.69 | 0.73 | 0.04 | 0.489 |
| Antenatal care with anti-malarial | 0.44 | 0.48 | 0.04 | 0.410 |
| Institutional delivery | 0.82 | 0.83 | 0.00 | 0.948 |
| Any postnatal care | 0.08 | 0.10 | 0.01 | 0.773 |
| Growth monitoring in past 6 months for children under 5 | 0.50 | 0.51 | -0.01 | 0.578 |
| Children aged 13-24 months with all basic vaccinations | 21.46 | 22.20 | 0.54 | 0.485 |

**Notes**: analysis of data from the household surveys. The difference between the two groups is estimated with multivariate regression models in which the outcome is regressed on the PBF treatment, controlling for randomization block (province). Standard errors are clustered at the health zone level.

## Additional results

Estimation of a large number of coefficients raises the concern of overemphasizing any individual significant result. The main way in which we address this in our analysis is with the meta-analysis approach used for estimation of average effect by domain. The tables below report the weights each individual outcome is given in the calculation of the meta-analysis coefficients, when data from health centers and hospitals is analyzed separately. In addition, we also calculate we calculate q-values following Benjamini-Hochberg (1995) and Anderson (2008), by domain, and compare these values to the estimated p-value of each individual regression. The results show that although the significance level of a few individual outcomes changes once accounting for the multiple hypothesis testing, the overall results do not change.

Table S7: Q-values and meta-analysis weights for outcomes at the health facility level

| **Variable** | **Health Centers** | | | | **Hospitals** | | | |
| --- | --- | --- | --- | --- | --- | --- | --- | --- |
|  | **p-value** | **q-value** | **Meta-analysis weight** |  | **p-value** | **q-value** | **Meta-analysis weight** |  |
| **Panel a: structural quality** | | | | | | | | |
| Basic equipment index | 0.020 | 0.052 | 24.649 |  | 0.586 | 1.000 | 56.901 |  |
| Essential medicine and consumables index | 0.732 | 0.445 | 20.465 |  | 0.227 | 1.000 | 18.123 |  |
| Vaccines index | 0.503 | 0.432 | 7.162 |  | 0.415 | 1.000 | 4.068 |  |
| Family planning products index | 0.000 | 0.001 | 12.202 |  | 0.958 | 1.000 | 5.733 |  |
| Infrastructure index | 0.205 | 0.258 | 21.571 |  | 0.350 | 1.000 | 9.214 |  |
| Infection prevention and control index | 0.144 | 0.239 | 13.951 |  | 0.839 | 1.000 | 5.961 |  |
| **Panel b: technical process quality** | | | | | | | | |
| Antenatal care score | 0.449 | 0.664 | 18.319 |  | 0.067 | 0.227 | 20.961 |  |
| IMCI: assessment score | 0.118 | 0.607 | 29.147 |  | 0.120 | 0.227 | 28.296 |  |
| IMCI: diagnosis score | 0.158 | 0.607 | 11.794 |  | 0.123 | 0.227 | 3.886 |  |
| IMCI: correct treatment | 0.330 | 0.607 | 5.422 |  | 0.354 | 0.227 | 7.087 |  |
| IMCI: no unnecessary treatment | 0.245 | 0.607 | 3.346 |  | 0.755 | 0.337 | 1.459 |  |
| Family planning consultation score | 0.001 | 0.006 | 8.556 |  | 0.225 | 0.227 | 9.574 |  |
| Delivery score | 0.285 | 0.607 | 11.789 |  | 0.097 | 0.227 | 15.869 |  |
| Postpartum care score | 0.287 | 0.607 | 7.859 |  | 0.026 | 0.227 | 4.835 |  |
| Newborn care score | 0.444 | 0.664 | 3.767 |  | 0.065 | 0.227 | 8.034 |  |
| **Panel c: non-technical process quality** | | | | | | | | |
| Antenatal care respect index | 0.426 | 0.831 | 8.457 |  | 0.683 | 1.000 | 5.302 |  |
| Antenatal care user satisfaction | 0.194 | 0.480 | 29.109 |  | 0.810 | 1.000 | 13.842 |  |
| Child curative care respect index | 0.753 | 1.000 | 5.457 |  | 0.096 | 1.000 | 9.798 |  |
| Child curative care user satisfaction | 0.974 | 1.000 | 16.811 |  | 0.835 | 1.000 | 13.342 |  |
| Family planning consultation respect index | 0.008 | 0.059 | 8.902 |  | 0.407 | 1.000 | 7.187 |  |
| Family planning consultation user satisfaction | 0.630 | 1.000 | 13.350 |  | 0.868 | 1.000 | 18.724 |  |
| Delivery care respect index | 0.071 | 0.269 | 17.914 |  | 0.794 | 1.000 | 31.805 |  |
| **Panel d: service fees (in Congolese Francs)** | | | | | | | | |
| ANC fee reported by facility | 0.017 | 0.076 | 17.375 |  | 0.642 | 1.000 | 13.000 |  |
| Delivery fee reported by facility | 0.068 | 0.098 | 3.911 |  | 0.307 | 1.000 | 2.991 |  |
| Family planning consultation fee reported by facility | 0.303 | 0.222 | 22.123 |  | 0.244 | 1.000 | 50.573 |  |
| Curative care fee reported by facility (not specific for child care) | 0.071 | 0.098 | 14.593 |  | 0.737 | 1.000 | 3.916 |  |
| ANC fees reported in exit interviews | 0.706 | 0.546 | 12.854 |  | 0.286 | 1.000 | 10.406 |  |
| Child curative care fees reported in exit interviews | 0.020 | 0.076 | 11.629 |  | 0.444 | 1.000 | 4.176 |  |
| Family planning consultation fee reported in exit interviews | 0.824 | 0.547 | 17.516 |  | 0.340 | 1.000 | 14.938 |  |
| **Panel e: fee policies and user satisfaction with fees** | | | | | | | | |
| Flat fees |  |  |  |  |  |  |  |  |
| Fees posted | 0.002 | 0.004 | 13.513 |  | 0.307 | 0.318 | 8.134 |  |
| Fee exemptions for poor users | 0.002 | 0.004 | 16.307 |  | 0.005 | 0.026 | 14.954 |  |
| Satisfaction with service affordability in antenatal care exit interviews | 0.000 | 0.001 | 18.206 |  | 0.608 | 0.575 | 12.643 |  |
| Satisfaction with service affordability in child care exit interviews | 0.010 | 0.008 | 18.417 |  | 0.008 | 0.026 | 36.282 |  |
| Satisfaction with service affordability in family planning exit interviews | 0.418 | 0.163 | 22.218 |  | 0.779 | 0.639 | 18.157 |  |
| **Panel f: facility management** | | | | | | | | |
| Protocols index | 0.010 | 0.016 | 29.064 |  | 0.114 | 0.130 | 31.227 |  |
| Reporting index | 0.053 | 0.037 | 43.648 |  | 0.276 | 0.162 | 39.772 |  |
| Posting of infection and control procedures index | 0.004 | 0.016 | 14.509 |  | 0.008 | 0.035 | 17.027 |  |
| Mechanism to Seek patients’ opinions | 0.306 | 0.083 | 12.778 |  | 0.070 | 0.117 | 11.973 |  |
| **Panel g: providers’ satisfaction** | | |  |  |  |  |  |  |
| Satisfied with information on own performance | 0.467 | 1.000 | 5.082 |  | 0.259 | 1.000 | 3.210 |  |
| Satisfied with level of autonomy | 0.182 | 1.000 | 6.836 |  | 0.976 | 1.000 | 5.384 |  |
| Satisfied with the relationship with the local facility committee | 0.738 | 1.000 | 6.740 |  | 0.192 | 1.000 | 4.860 |  |
| Satisfied with support from supervisor | 0.307 | 1.000 | 6.411 |  | 0.181 | 1.000 | 4.778 |  |
| Satisfied with recognition received from supervisor | 0.558 | 1.000 | 8.685 |  | 0.294 | 1.000 | 9.036 |  |
| Satisfied with reward received from work | 0.674 | 1.000 | 3.655 |  | 0.247 | 1.000 | 2.622 |  |
| Satisfied with ability to use skills | 0.347 | 1.000 | 8.366 |  | 0.423 | 1.000 | 4.646 |  |
| Satisfied with training opportunities | 0.790 | 1.000 | 3.180 |  | 0.551 | 1.000 | 3.684 |  |
| Satisfied with security at the facility | 0.576 | 1.000 | 8.160 |  | 0.739 | 1.000 | 8.362 |  |
| Satisfied with work conditions | 0.495 | 1.000 | 3.860 |  | 0.696 | 1.000 | 3.781 |  |
| Satisfied with leave | 0.499 | 1.000 | 3.724 |  | 0.132 | 1.000 | 8.349 |  |
| Satisfied with work hours | 0.607 | 1.000 | 3.647 |  | 0.985 | 1.000 | 5.971 |  |
| Satisfied with teamwork | 0.569 | 1.000 | 15.884 |  | 0.694 | 1.000 | 13.243 |  |
| Satisfied with relationship with facility management | 0.244 | 1.000 | 8.855 |  | 0.727 | 1.000 | 13.058 |  |
| Satisfied with income overall | 0.928 | 1.000 | 3.224 |  | 0.534 | 1.000 | 4.579 |  |
| Satisfied with potential for promotion | 0.897 | 1.000 | 3.691 |  | 0.529 | 1.000 | 4.437 |  |

Table S8: Q-values and meta-analysis weights for incentivized services

|  | **p-value** | **q-value** | **Meta-analysis weight** |  |
| --- | --- | --- | --- | --- |
| Early antenatal care initiation (first trimester) | 0.002 | 0.015 | 9.436 |  |
| At least 4 antenatal care visits during last pregnancy | 0.476 | 0.914 | 2.443 |  |
| Antenatal care with tetanus shot | 0.212 | 0.591 | 7.157 |  |
| Antenatal care with anti-malarial | 0.878 | 1.000 | 3.257 |  |
| Institutional delivery | 0.210 | 0.591 | 11.309 |  |
| Any postnatal care | 0.500 | 0.914 | 2.770 |  |
| Modern family planning method among women aged 15-49 | 0.007 | 0.027 | 34.515 |  |
| Growth monitoring in past 6 months for children under 5 | 0.445 | 0.914 | 27.482 |  |
| Children aged 13-24 months with all basic vaccinations | 0.993 | 1.000 | 1.631 |  |

The figures below show the results of the pooled analysis of data from health centers and hospitals. They present the estimated impact coefficients for the individual outcomes as well as the weights assigned for each outcome in the meta-analysis.

Figure S2: pooled analysis of structural quality outcomes


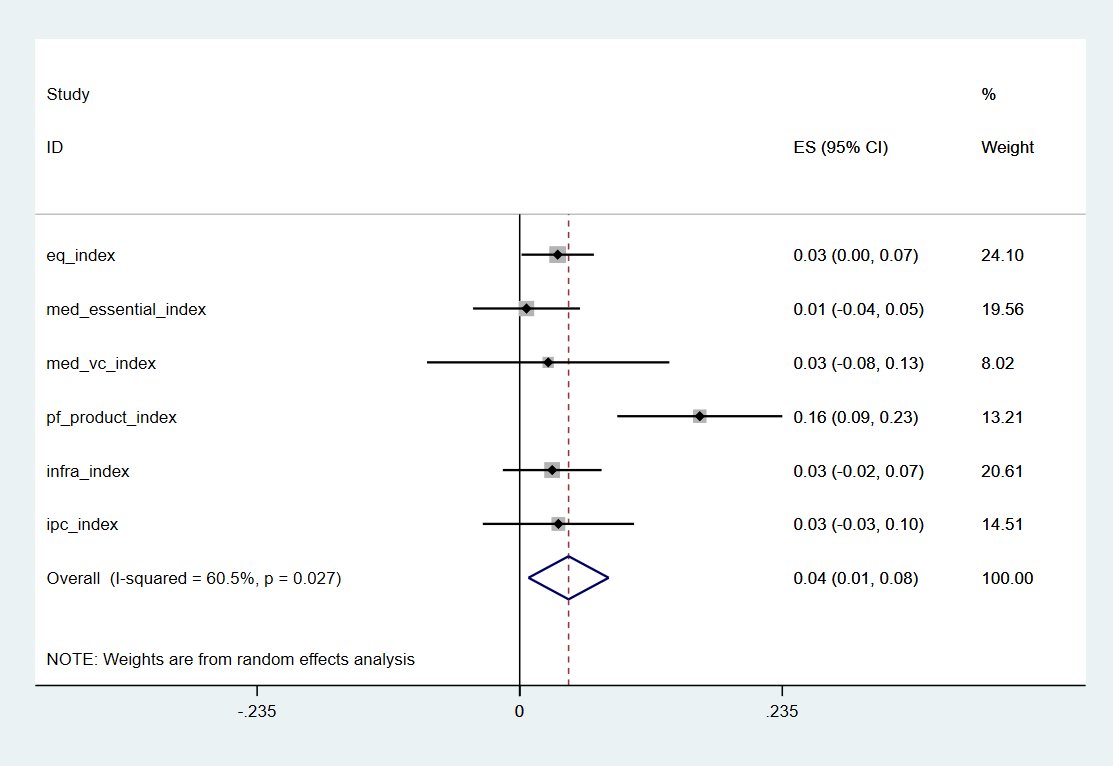


Figure S3: pooled analysis of technical process quality outcomes


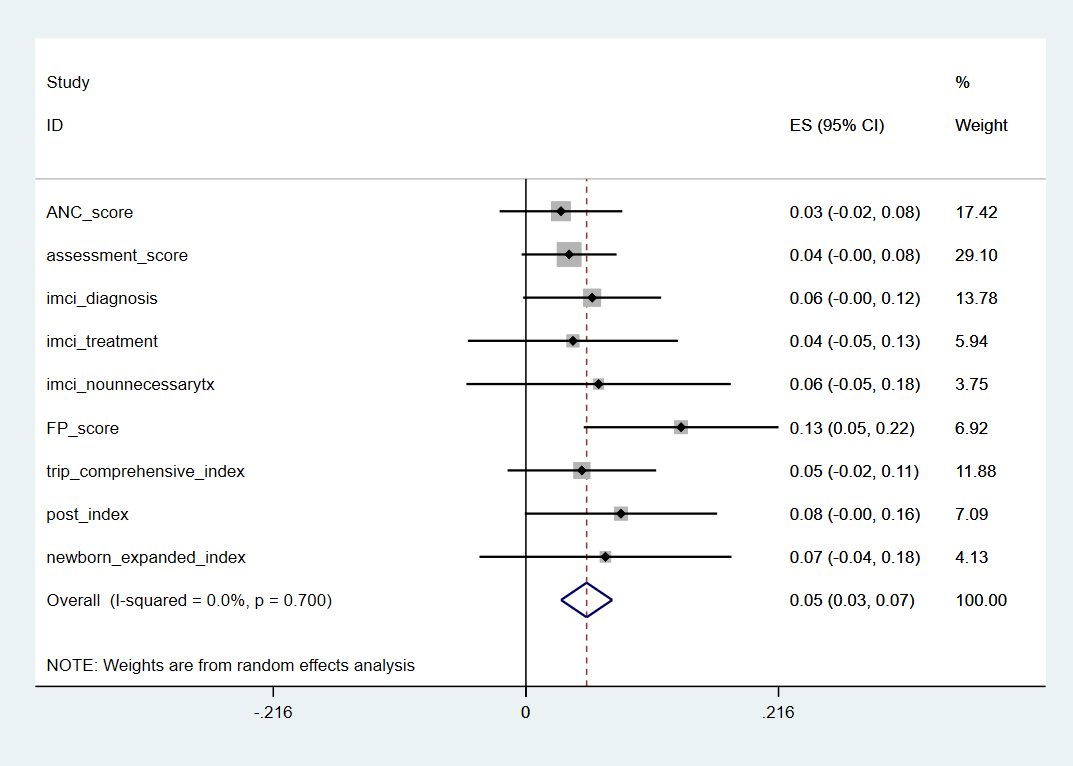


Figure S4: pooled analysis of non-technical quality outcomes


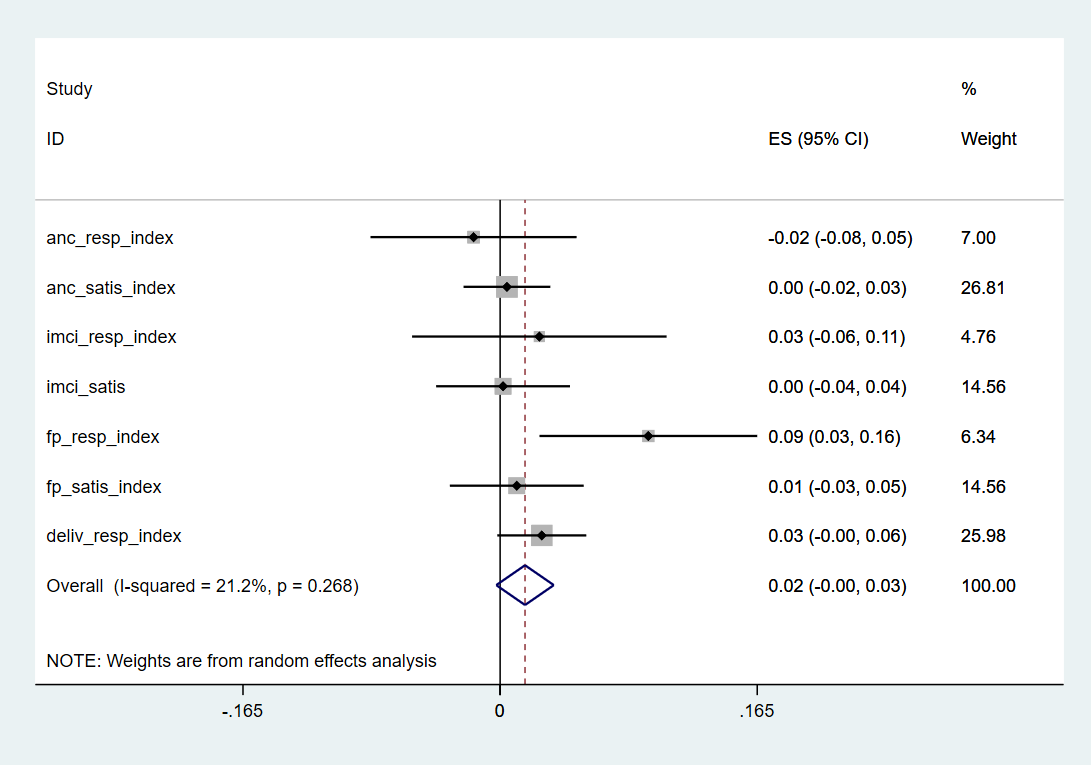


Figure S5: pooled analysis of service fees outcomes


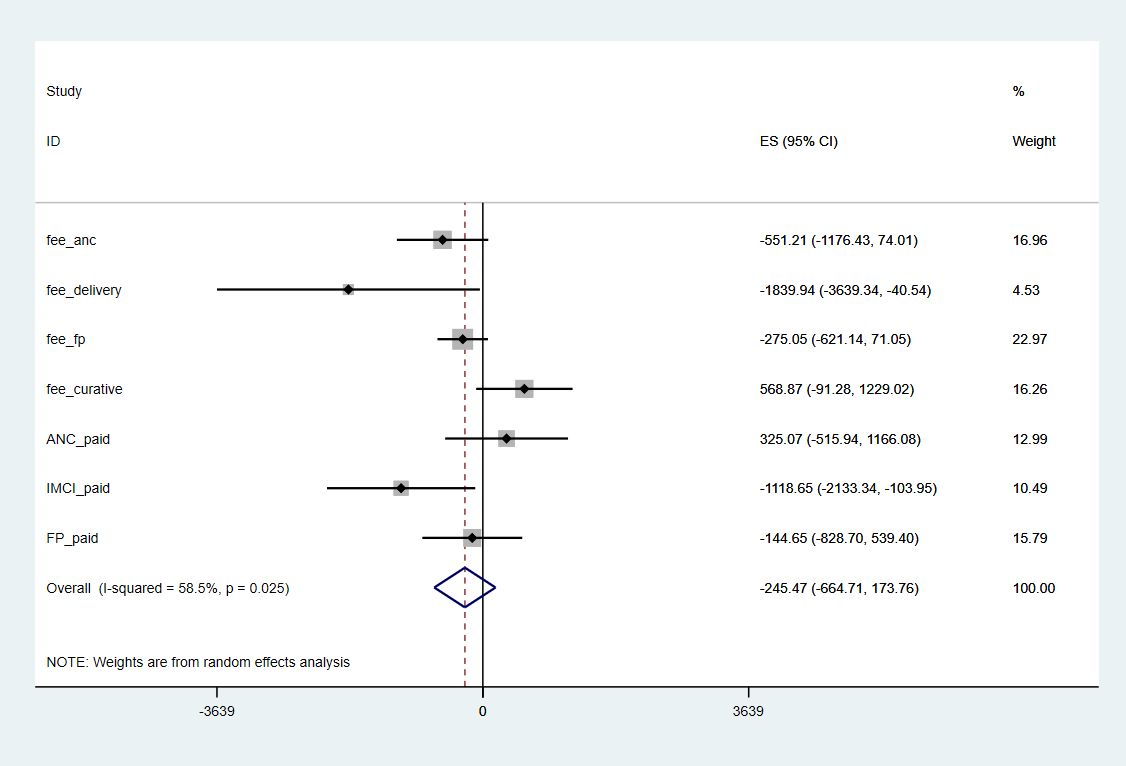


Figure S6: pooled analysis of fee policies and user satisfaction with affordability outcomes


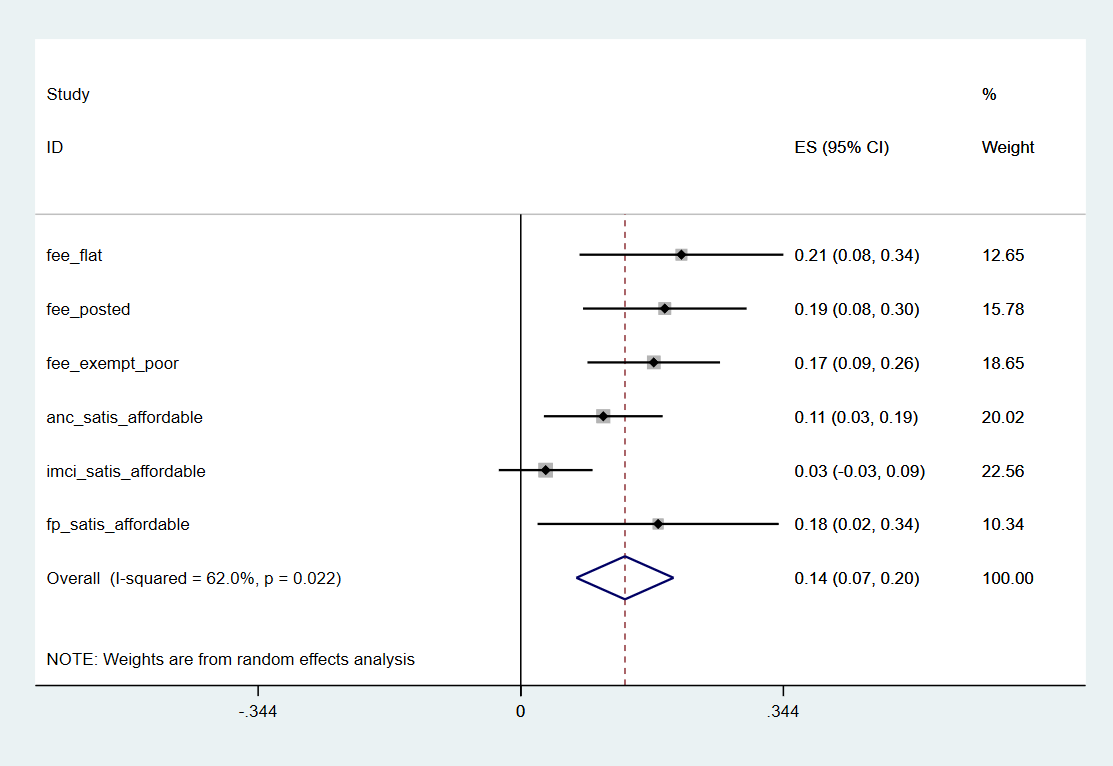


Figure S7: pooled analysis of facility management outcomes


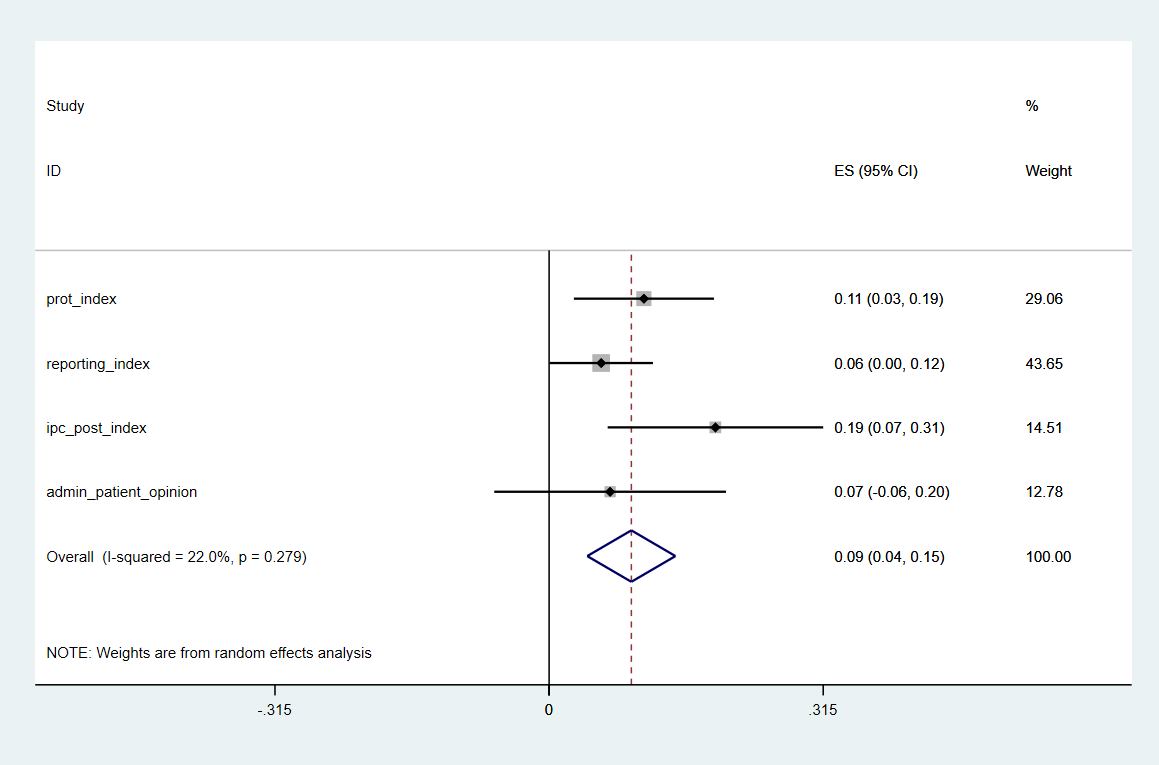


Figure S8: pooled analysis of providers’ satisfaction outcomes


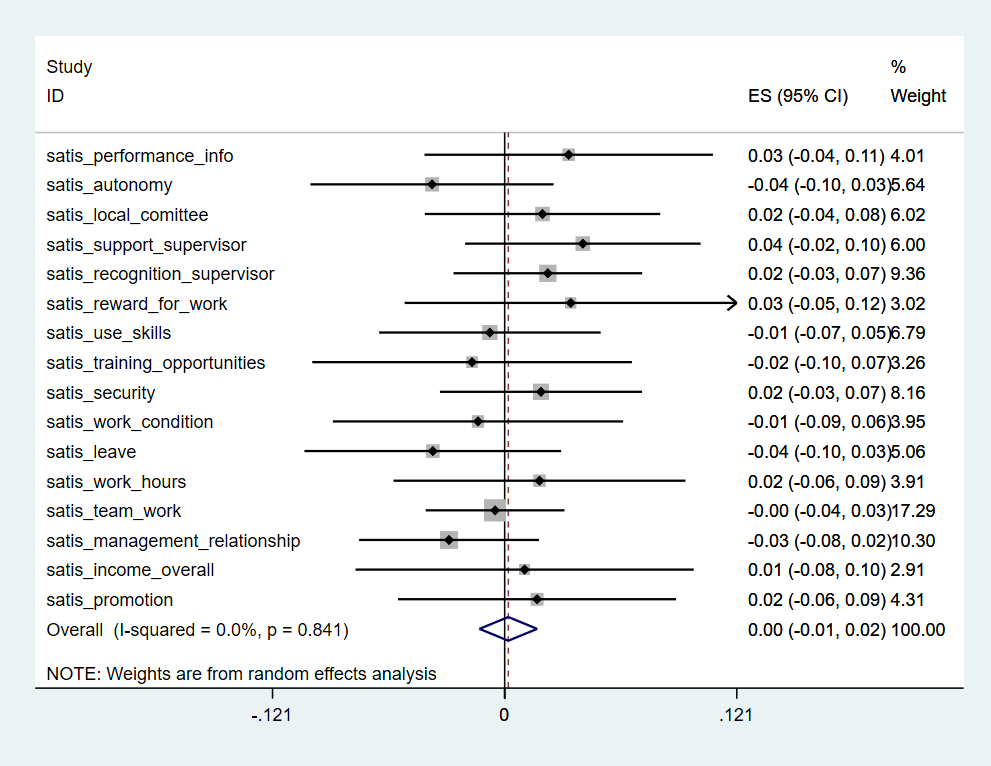

Supplement: Supplementary file 1 — Additional file 1: Information on the DRC performance-based financing program, the randomization, the data collection, the definition of the outcome measures, the statistical methods, and provides additional results. Table S1. PBF quantity indicators and corresponding relative weights - Minimum Package of Activities (Health centers). Table S2. PBF quantity indicators and corresponding relative weights - Minimum Package of Activities (Hospitals). Table S3. Quality checklist components and weights for the minimum package of activities (health centers). Table S4. Quality checklist components and weights for the complementary package of activities (hospitals). Figure S1. Spatial location of study areas and results of the intervention randomization. Table S5. Baseline balance of outcomes measured through health facility assessments. Table S6. Baseline balance of outcomes measured through household surveys. Table S7. Q-values and meta-analysis weights for outcomes at the health facility level. Table S8. Q-values and meta-analysis weights for incentivized services. Figure S2. Pooled analysis of structural quality outcomes. Figure S3. Pooled analysis of technical process quality outcomes. Figure S4. Pooled analysis of non-technical quality outcomes. Figure S5. Pooled analysis of service fees outcomes. Figure S6. Pooled analysis of fee policies and user satisfaction with affordability outcomes Figure S7. Pooled analysis of facility management outcomes. Figure S8. Pooled analysis of providers’ satisfaction outcomes. [file 12916_2023_3062_MOESM1_ESM.docx]
